# Microbial Keratinase: Next Generation Green Catalyst and Prospective Applications

**DOI:** 10.3389/fmicb.2020.580164

**Published:** 2020-12-18

**Authors:** Nonso E. Nnolim, Chibuike C. Udenigwe, Anthony I. Okoh, Uchechukwu U. Nwodo

**Affiliations:** ^1^SAMRC Microbial Water Quality Monitoring Centre, University of Fort Hare, Alice, South Africa; ^2^Applied and Environmental Microbiology Research Group (AEMREG), Department of Biochemistry and Microbiology, University of Fort Hare, Alice, South Africa; ^3^School of Nutrition Sciences, Faculty of Health Sciences, University of Ottawa, Ottawa, ON, Canada

**Keywords:** microbial keratinase, valorization, keratin-rich wastes, biodegradation, amino acids, enzyme technology

## Abstract

The search for novel renewable products over synthetics hallmarked this decade and those of the recent past. Most economies that are prospecting on biodiversity for improved bio-economy favor renewable resources over synthetics for the potential opportunity they hold. However, this field is still nascent as the bulk of the available resources are non-renewable based. Microbial metabolites, emphasis on secondary metabolites, are viable alternatives; nonetheless, vast microbial resources remain under-exploited; thus, the need for a continuum in the search for new products or bio-modifying existing products for novel functions through an efficient approach. Environmental distress syndrome has been identified as a factor that influences the emergence of genetic diversity in prokaryotes. Still, the process of how the change comes about is poorly understood. The emergence of new traits may present a high prospect for the industrially viable organism. Microbial enzymes have prominence in the bio-economic space, and proteases account for about sixty percent of all enzyme market. Microbial keratinases are versatile proteases which are continuously gaining momentum in biotechnology owing to their effective bio-conversion of recalcitrant keratin-rich wastes and sustainable implementation of cleaner production. Keratinase-assisted biodegradation of keratinous materials has revitalized the prospects for the utilization of cost-effective agro-industrial wastes, as readily available substrates, for the production of high-value products including amino acids and bioactive peptides. This review presented an overview of keratin structural complexity, the potential mechanism of keratin biodegradation, and the environmental impact of keratinous wastes. Equally, it discussed microbial keratinase; vis-à-vis sources, production, and functional properties with considerable emphasis on the ecological implication of microbial producers and catalytic tendency improvement strategies. Keratinase applications and prospective high-end use, including animal hide processing, detergent formulation, cosmetics, livestock feed, and organic fertilizer production, were also articulated.

## Introduction

Keratin, a fibrous and structural polypeptide, is highly recalcitrant to degradation by common proteolytic processes due to its molecular architecture. Putting nature’s biomass abundance into perspective, keratin has been ranked the third most abundant and is only surpassed by chitin and cellulose (Hossain et al., 2007). The orientation and interactions inherent in the bonds found in keratin have been reported to be the leading cause of its recalcitrance to degradation (Brandelli, 2008; Bach et al., 2011). Keratin is an intrinsic part of the epidermis and appendages, including hair, feathers, nails, horns, hooves, scales, and wool. Mechanical support and functional protection are some of the physiological functions provided by keratin (Gupta and Ramnani, 2006; Jaouadi et al., 2013). Tight packing of individual α-helix or β-sheet motifs of the polymer chains into a supercoiled strand confers mechanical stability to the resultant structure (Onifade et al., 1998). Also, keratin is categorized as hard or soft in relation to the sulfur group content, site of occurrence, and functional involvement of the polypeptide (Li, 2019). The keratin in nails, feathers, horns, and hooves are said to be hard due to their involvement in structural and protective roles against predation and abiotic factors, hence the toughness (Wang B. et al., 2016). However, soft keratins in the skin and callus have fewer disulfide bonds that allow the pliability of their structures (Schrooyen et al., 2001).

Agro-industrial based keratinous wastes constitute an ecosystem nuisance, and top on the list are feathers and hair arising from poultry and leather processing industries that strive to meet the demand of the burgeoning human population (Kang et al., 2018; Kalaikumari et al., 2019). Degradation of these wastes by classic proteases such as papain, pepsin, and trypsin is considerably tough (Thankaswamy et al., 2018). The molecular conformations of the crude protein contents of the keratinous materials, such as intense disulfide bond cross-linkages, hydrogen bonds and hydrophobic interactions improve the structural stability and prevent the proteolytic hydrolysis (Sangali and Brandelli, 2000). Consequently, the wastes continue to constitute environmental problems, which may pose public health problems (Guo et al., 2020). In the past, keratinous wastes and other animal carcasses were harmoniously baked, pulverized, and subsequently utilized as a nutritional supplement of domestic animal feeds (Sapkota et al., 2007). However, the danger posed by prion disease impeded the use of poultry and animal waste recycling in animal husbandry (Tsiroulnikov et al., 2004).

Microbial proteolytic enzymes capable of effectively degrading keratin are referred to as keratinases (3.4.21/24/99) and are considered highly valuable in many biotechnological processes due to their robustness in management of recalcitrant substrates and appreciable stability under extreme conditions (Abdel-fattah et al., 2015).

Conventionally processed protein-rich feeds of keratin origin are not typically metabolized by animals due to the absence of the redox system that hydrolyses the disulfide bonds of the polypeptides (Wang et al., 2006; Lange et al., 2016). Hence, the nutritive value of keratin-based feed-products needs to be improved by enhancing protein digestibility and nutrient availability (Nnolim et al., 2020a). Keratinase-mediated keratin solubilization offers a solution toward improving the nutritional qualities of the keratin-based feed-products in the fermentative process, which may be submerged or solid state (Łaba and Szczekała, 2013). Microbial keratinase-assisted solubilization of keratin is important in the bio-economy innovation value chain. It enables a sustainable waste management, while promoting the valorization of intractable waste bio-resources into value-added products for the green economy (De Oliveira Martinez et al., 2020). However, the mechanisms of action leading to the complete breakdown of keratinous substrates by keratinolytic proteases are not yet fully elucidated. It is conceived that keratinous materials are enzymatically dismembered through the reduction of the disulfide bridges and cleavage of peptide bonds (Stiborova et al., 2016). This review highlights the keratinase sources and functional properties, ecological implication of keratinase producers, environmental impact of keratinous wastes, keratinase applications, and protein structure modification to enhance the catalytic efficiency of keratinase.

## Microbial Keratinase and Functional Properties

Keratinolytic proteases have been reported to be associated with an array of microbes isolated from a diverse milieu of the ecosystem. Essentially, bacteria and fungi have been identified as good decomposers of keratinous substrates, which they accomplish by extracellular production of keratinolytic enzymes.

### Bacterial Sources

Keratinase production potential has been reported within the bacterial genus of Bacillus, Vibrio, Chryseobacterium, Brevibacillus, Pseudomonas, Serratia, Fervidobacterium, Microbacterium, Aeromonas, Burkholderia, Stenotrophomonas, Rhodococcus, Geobacillus Amycolatopsis, Meiothermus, and Paenibacillus (Williams et al., 1990; Friedrich and Antranikian, 1996; Sangali and Brandelli, 2000; Yamamura et al., 2002; Riffel and Brandelli, 2006; Bach et al., 2011; Kuo et al., 2012; Fang et al., 2013; Jaouadi et al., 2013; Paul et al., 2014b; Stiborova et al., 2016; Abdel-Fattah et al., 2018; Falco et al., 2019; Alahyaribeik et al., 2020; Nnolim et al., 2020a,b). Keratinase production by Actinobacteria, particularly Streptomyces, Arthrobacter, Bevibacterium, and Nocardiopsis, have been reported (Mitsuiki et al., 2006; Jaouadi et al., 2010; Pereira et al., 2014; Gong et al., 2015; Barman et al., 2017; Thankaswamy et al., 2018).

### Fungal Sources

Keratinolytic fungi have been reported as the natural colonizers of keratinous substrates and play a major role in the natural hydrolysis of keratinized tissues (Bohacz et al., 2020). Aspergillus, Paecilomyces, Doratomyces, Trichoderma, Fusarium, Acremonium, Onygena, Cladosporium, Microsporum, Lichtheimia, Chrysosporium, Aphanoascus, Trichophyton, and Scopulariopsis are among the reported keratinolytic fungi (Gradišar et al., 2000, 2005; Sharma et al., 2011; Paul et al., 2014a; Sousa et al., 2015; Bagewadi et al., 2018; Abirami et al., 2020; Bohacz et al., 2020). Among the fungal strains, keratinolytic activity has been extensively reported within the confines of the dermatophytes. Keratinolytic property of this group could represent the pathogenic tendencies and has been implicated in mycosis of human and animal skin (Korniłłowicz-Kowalska and Bohacz, 2011).

Even though keratinase potential has been reported for different microbial species autochthonous to variable ecological niches, the large-scale production has been a challenge from the commercial perspective (Daroit and Brandelli, 2014). Inherent cell precursors, culture conditions, and nutrient availability are among the significant factors that influence microbial metabolites production (Parra et al., 2005). The genetic diversity of keratinolytic microorganisms affects the arbitrary selection of the physico-chemical fermentation conditions (Arokiyaraj et al., 2019). Additionally, the prolonged fermentation period for the keratinase production by most wild microbial producers affects keratinase yield and sustainability (Fang et al., 2019). Hence, this demands developing industrially viable strains to enhance productivity beyond the bench scale. The nature of nutrient in the cultivation medium plays important roles in the expression of genes coding for the metabolites of interest. Generally, keratinases are secreted extracellularly by the microbial producer in the presence of keratinous substrate; however, cell-bound (Riessen and Antranikian, 2001; Nam et al., 2002) and internally produced keratinases (Onifade et al., 1998) have also been reported. The expression of keratinase encoding genes and activation of redox systems are thought to be induced by the presence of keratinous substrates, which are bioconverted to essential nutritional factors for maintaining microbial homeostasis (Thankaswamy et al., 2018). Alkalinization of the fermentation medium is a crucial factor in keratinous biomass degradation; it is thought that alkalinization softens the keratinous substrate, thus improving the process of sulfitolysis and proteolysis (Kunert, 2000; Ramnani et al., 2005).

### Biochemical Properties of Keratinase

Understanding the biochemical properties of microbial proteases is vital for the potential application of the enzymes in the bio-industry. The properties of keratinolytic proteases are dependent on the producing organism (Selvam and Vishnupriya, 2012). Here, we discuss some of these properties, including pH and temperature optima, molecular weight, substrate specificity as well as the effects of reducing agents, inhibitors, surfactants, metal ions, and chemical solvents on the catalytic properties of keratinases. Some general properties of selected keratinolytic proteases are presented in Table 1. Majority of keratinolytic proteases are catalytically active within neutral to alkaline pH with optima values between pH 7.0 and 9.0 (Gradišar et al., 2000; Bach et al., 2011; Kuo et al., 2012; Jaouadi et al., 2013; Bose et al., 2014; Akhter et al., 2020). However, a few extremely alkalophilic keratinases with optimum pH between 10 and 13 were previously reported (Friedrich and Antranikian, 1996; Letourneau et al., 1998; Mitsuiki et al., 2006; Jaouadi et al., 2008, 2010; Tatineni et al., 2008; Benkiar et al., 2013; Paul et al., 2014b; Gong et al., 2015; Tian et al., 2019). The optimum temperature reported for the activity of several microbial keratinases was between 37 and 65°C (Hossain et al., 2007; Bach et al., 2011; Jaouadi et al., 2013; Wu et al., 2017; Hassan et al., 2020b), whereas thermophilic keratinolytic proteases already characterized function optimally between 70 and 100°C (Nam et al., 2002; Jaouadi et al., 2010; Kuo et al., 2012; Benkiar et al., 2013; Paul et al., 2014b; Gong et al., 2015). Many keratinases, irrespective of the sources, have been found to be catalytically active and stable over a temperature and pH range of 20 to 100°C, and 4 to 13, respectively (Jaouadi et al., 2008, 2010; Paul et al., 2014b; Gong et al., 2015; Jin et al., 2019).

**TABLE 1:** Summary of biochemical properties of some selected microbial keratinases and their potential applications.

**Microbial source**	**Protease type**	**Activity substrate**	**Optimum temp. (°C)**	**Optimum pH**	**^#^Stabilizer/Promoter**	*MW (kDa)	**Application**	**References**
**Bacterial source**								
*Actinomadura keratinilytica* Cpt29	Serine	Keratin azure	70	10	Mn^2+^	29.23	Feather degradation	Habbeche et al., 2014
*Bacillus circulans* DZ100	Serine	Keratin azure	85	12.5	Ca^2+^, Mn^2+^, and Mg^2+^	32.02	Keratin degradation, Detergent formulation	Benkiar et al., 2013
*Bacillus tequilensis* hsTKB2	Serine	Keratin azure	70	10.5	Toluene, Mn^2+^	59.89	Detergent formulation	Paul et al., 2014c
*Bacillus pseudofirmus* FA30-01	Serine	Azokeratin	60	8.8–10.3	Mg^2+^, Co^2+^, and Zn^2+^	29.29	Feather degradation	Kojima et al., 2006
*Bacillus licheniformis*	Serine	Keratin azure	60	8.5	Mn^2+^, Al^3+^, and Ca^2+^	35	Keratin hydrolysis	Suntornsuk et al., 2005
*Bacillus licheniformis* ER-15	Serine	Feather	70	11	β-ME	58	Feather degradation, Hide dehairing	Tiwary and Gupta, 2010
*Bacillus subtilis* PF1	−	Casein	60	9	Ca^2+^, Trition X-100, DMSO	−	Detergent formulation	Bhange et al., 2016
*Bacillus* sp. CL18	Serine	Azocasein	55	8	Ca^2+^, Mg^2+^, Trition X-100, DMSO	−	Keratin hydrolysis	Rieger et al., 2017
*Bacillus* sp. AD-W	Serine	Keratin azure	50	10	Mn^2+^, DTT	39	Keratin hydrolysis	Gegeckas et al., 2018
*Bacillus* sp. AD-AA3	Serine	Keratin azure	50	8	DTT, β-ME	29	Keratin hydrolysis	Gegeckas et al., 2018
*Bacillus subtilis* FTC02PR1	Serine	Azokeratin	60	6–11	SDS, Mn^2+^	30	Feather degradation	Ferrareze et al., 2016
*Bacillus licheniformis* ALW1	−	Keratin	65	8	−	−	Feather degradation	Abdel-Fattah et al., 2018
*Bacillus haynesii* ALW2	−	Keratin	70	8–9	−	−	Hide dehairing	Emran et al., 2020
*Bacillus amyloliquefaciens* S13	Serine	Keratin azure	50	6.5	Ca^2+^, Mg^2+^, and Zn^2+^	28	Feather degradation, Hide unhairing	Hamiche et al., 2019
*Bacillus amyloliquefaciens* S13	Serine	Keratin azure	60	8	Ca^2+^, Mg^2+^, and Mn^2+^	47	Feather degradation, Hide unhairing	Hamiche et al., 2019
*Bacillus thuringiensis* MT1	Metallo	Keratin	50	9	Ba^2+^, Ca^2+^, Mg^2+^, and Mn^2+^	80	Hair degradation	Hassan et al., 2020b
*Bacillus subtilis* SCK6	Serine	Keratin	60	10	Cu^2+^, Co^2+^	30.95	Skin dehairing	Tian et al., 2019
*Brevibacillus brevis* US575	Serine	Keratin azure	40	8	Ca^2+^	29.12	Hide dehairing	Jaouadi et al., 2013
*Brevibacillus parabrevis*	Serine	Keratin	60	8	Na^+^, Ca^2+^, Triton X-100, Tween-40	28	Hide dehairing	Zhang et al., 2016
*Brevibacterium luteolum*	−	Keratin azure	30	10	−	−	Hair degradation	Thankaswamy et al., 2018
*Caldicoprobacter algeriensis*	Serine	Keratin azure	50	7	Sn^2+^, Ba^2+^, Ca^2+^, Mg^2+^, and Mn^2+^	33.25	Hide dehairing	Bouacem et al., 2016
*Chryseobacterium* L99 sp. nov.	Serine	Keratin azure	40	8	K^+^, Zn^2+^, and Co^2+^	33	Wool treatment	Lv et al., 2010
*Fervidobacterium islandicum*	Metallo	−	80	7	Co^2+^	−	Feather degradation	Lee et al., 2015
*Fervidobacterium pennavorans*	Serine	Casein	80	10	−	130	Feather degradation	Friedrich and Antranikian, 1996
*Meiothermus* sp. 140	Serine	Keratin azure	70	8	Ethanol, DMSO	76	Feather degradation	Kuo et al., 2012
*Meiothermus taiwanensis* WR-220	−	Chicken feather	65	10	−	30	Feather degradation	Wu et al., 2017
*Microbacterium sp. kr10*	Metallo	Azocasein	50	7.5	Zn^2+^ and Mg^2+^	42	Feather degradation	Thys and Brandelli, 2006
*Serratia marcescens* P3	Metallo	Azokeratin	40–45	6.5	−	53	Skin dehairing	Bach et al., 2012
*Streptomyces* sp.AB1	Serine	Keratin azure	75	11.5	Mg^2+^	29.85	Feather degradation	Jaouadi et al., 2010
*Streptomyces* sp. S.K_1__–__02_	Serine-metallo	Keratin azure	70	10	Ca^2+^ and Mg^2+^	−	Keratin hydrolysis	Letourneau et al., 1998
*Streptomyces* sp. S7	Serine-metallo	Keratin azure	45	11	Ca^2+^	44	−	Tatineni et al., 2008
*Streptomyces aureofaciens* K13	Serine-metallo	Keratin	75	12	Cu^2+^, Mn^2+^, and SDS	46	Keratin hydrolysis, Detergent formulation	Gong et al., 2015
**Fungal source**								
*Aspergillus fumigatus* TKF1	−	Keratin	50	6	−	24.3	Feather degradation	Paul et al., 2014a
*Aspergillus flavus* K-03	Serine	Keratin azure	40	8	β-ME, Hg^2+^, Fe^2+^, and Mn^2+^	31	−	Kim, 2007
*Aspergillus sulphureus*	−	Keratin	35	10	−	−	−	Sousa et al., 2015
*Doratomyces microspores*	Serine	Keratin	50	8–9	−	33	−	Gradišar et al., 2000
*Microsporum canis*	Metallo	Azocoll	50	8	−	43.5	−	Brouta et al., 2001
*Onygena corvina*	Serine	Keratin azure	40–60	6–11	Fe^2+^ and Ca^2+^	35	Feather degradation	Huang et al., 2015
*Scopulariopsis brevicaulis*	Serine	Chicken feather	40	8	Ca^2+^	75	Skin dehairing	Anbu et al., 2005
*Trichophyton sp.* HA-2	Serine	Chicken feather	40	7.8	−	34	−	Anbu et al., 2008

^#^β-ME, β-mercaptoethanol; DMSO, dimethyl sulfoxide; DTT, dithiothreitol; SDS, sodium dodecyl sulfate. *MW, molecular weight.

An overview of various reports on keratinolytic proteases of microbial sources shows different molecular weight range from 17 to 240 kDa (Friedrich and Antranikian, 1996; Bressollier et al., 1999; Nam et al., 2002; Bernal et al., 2006; Mitsuiki et al., 2006; Kim, 2007; Tatineni et al., 2008; Benkiar et al., 2013; Fang et al., 2013; Jaouadi et al., 2013; Hamiche et al., 2019; Hassan et al., 2020b). Keratinases from *S. maltophilia* BBE11-1 (Fang et al., 2013) and *S. albidoflavus* (Bressollier et al., 1999) have so far been documented to have low molecular weights of 17 and 18 kDa, respectively. In contrast, the keratinase produced by *K. rosea* was shown to have molecular weight as high as 240 kDa (Bernal et al., 2006). Generally, the majority of the documented keratinases are monomeric enzymes with molecular weights of ≤58 kDa (Table 1). However, a novel dimeric keratinolytic protease of 58 kDa with subunit molecular weights of 30 and 28 kDa has been reported (Tiwary and Gupta, 2010). Multimeric microbial keratinases have also been described (Brandelli et al., 2010).

Keratinolytic proteases generally belong to the serine or metallo class of peptidases regardless of the microbial source. The inhibition by diisopropyl fluorophosphate (DFP) or phenylmethanesulfonyl fluoride (PMSF) indicates their serine protease catalytic mechanism, while inhibition by ethylenediaminetetraacetic acid (EDTA) or 1,10-phenanthroline suggests their type as metalloproteases. EDTA inhibits keratinase of the metalloprotease class through the chelation of the metal ions that are involved in enzyme catalysis (Riffel et al., 2003). Partial inhibition of the keratinolytic enzymes by either EDTA or PMSF was previously reported (Letourneau et al., 1998; Kim, 2007; Tatineni et al., 2008; Gong et al., 2015), which might be an indication of the presence of mixed keratinolytic proteases.

The importance of cations such as Ca^2+^, Mg^2+^, Co^2+^, Ba^2+^, K^+^, Fe^2+^, Ni^2+^, Mn^2+^, and Li^+^ as stabilizing agents or activators of keratinases has been extensively reported (Bockle et al., 1995; Riessen and Antranikian, 2001; Nam et al., 2002; Tatineni et al., 2008; Jaouadi et al., 2010; Gong et al., 2015; Hamiche et al., 2019). Conversely, the negative impact of some metal ions including Pb^2+^, Hg^2+^, Cu^2+^, Fe^3+^, Ni^2+^, and Cd^2+^ on keratinase activity due to toxic insults has been previously observed (Lin et al., 2009; Benkiar et al., 2013; Su et al., 2017; Gegeckas et al., 2018). Usually, keratinase activities are inhibited, stabilized, or enhanced in a solution that contains surfactants, reducing agents, or solvents. These variable behaviors of keratinases toward chemical agents are attributable to the nature of their side chains/interaction patterns which allosterically regulate the structural orientation and biocatalytic efficiency of the enzymes (Fang et al., 2016). The activity of the alkaline keratinase from *S. aureofaciens* K13 was enhanced when incubated with SDS, Tween-80 or Triton-X100 (Gong et al., 2015). A similar result was obtained with serine keratinase from *B. licheniformis* ER-15 where enzymatic activity increased by 6.25 folds when supplemented with 5 mM mercaptoethanol (Tiwary and Gupta, 2010). This stability tendency of keratinolytic proteases in laboratory-based chemical agents has projected their candidacy as robust additives in industrial processes (Reddy et al., 2017; Emran et al., 2020).

Keratinolytic protease exhibits a broad specificity toward protein-containing natural and synthetic substrates. However, the existence of some discrepancies in substrate specificity for different microbial keratinases may be attributed to the isolation technique and genetic diversity of their sources (Onifade et al., 1998). The specificity of proteases to substrates is proposed to be group dependent, and it is mostly determined by the predominated sequence residues at the C-terminal (P1) and/or N-terminal (P1’) close to the peptide bond to be hydrolyzed (Rawlings, 2016). The insolubility of some proteinaceous substrates could be prompted by inherent inter- or intra-molecular forces such as disulfide bonds that confer mechanical stability to some structural proteins; hence, accessibility of peptide bonds by classical proteases remains impossible (Navone and Speight, 2018). Keratinases, on the other hand, have been naturally endowed with hydrolytic potentials for both soluble and insoluble proteins, and have been adopted, widely as promising candidates in many biotechnological processes (Hassan et al., 2020a). Yamamura et al. (2002) reported that the catalytic efficiencies established for subtilisin Carlsberg and proteinase K toward synthetic substrates were similar to those of protease D-1 from *Stenotrophomonas* sp. toward the hydrolysis of N-succinyl-L-alanyl-L-alanyl-L-prolyl-L-phenylalanine 4-nitroanilide and N-succinyl-L-alanyl-L-alanyl-L-alanine 4-nitroanilide, respectively. Moreover, *Stenotrophomonas* sp. protease D-1 hydrolyzed the rigid proteins (keratins, collagen, and elastin) poorly, compared to the soluble proteins (hemoglobin, bovine serum albumin, and casein). Sangali and Brandelli (2000) observed that the keratinase produced by *Vibrio* sp. kr2 was active toward azokeratin, azocasein, benzoyl-arginine p-nitroanilide, and Alanyl-L-Alanine p-nitroanilide as substrates, and this is an indication that the keratinase produced by strain kr2 may have the potential for different industrial processes and application.

## Structural Complexity of Keratin

**FIGURE 1:**
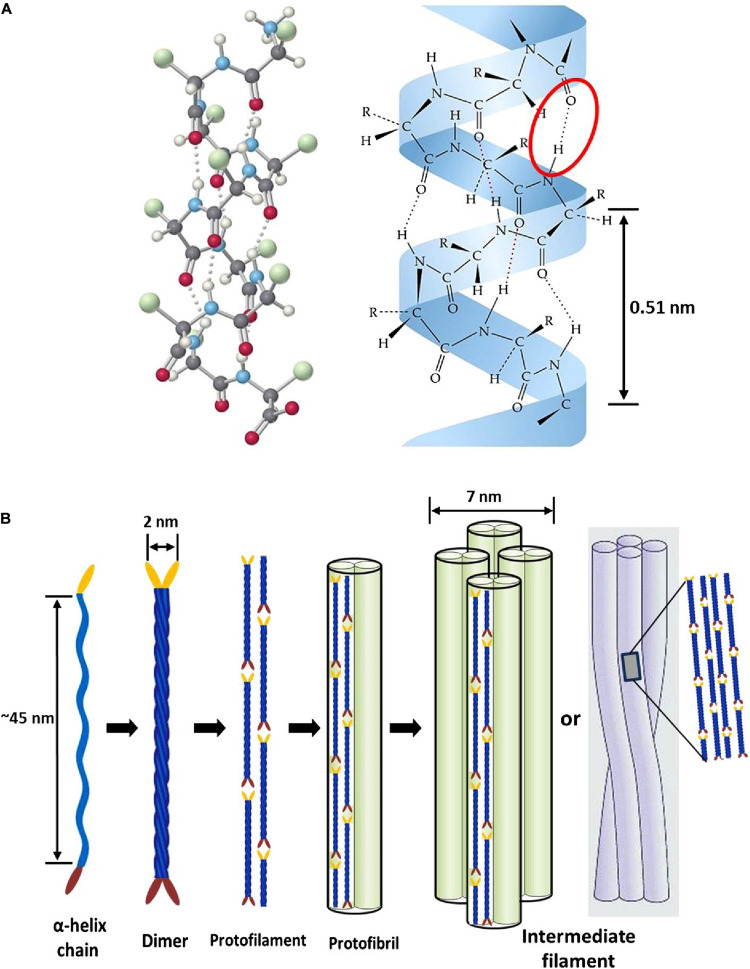
Intermediate filament structure of α-keratin: **(A)** ball and stick model of a typical protein chain with helical configuration, which is stabilized by many forces; however, the densely populated intra-molecular hydrogen bonds (red oval shape) mostly determine the helicity of the shape with 0.5 nm pitch; **(B)** schematic representation showing processes of intermediate filament formation. The association of two α-helices (45 nm long) produces left-handed dimeric- coils with diameter of 2 nm; the disulfide composition of these moieties initiate end to end aggregation and side-by-side staggering to produce protofilament; two protofilaments combine laterally into protofibril; lastly, the association of four protofibrils constitute the helical intermediate filaments with a diameter of 7 nm. Reproduced from Wang B. et al. (2016) with permission (permission number: 4380240601835).

**FIGURE 2:**
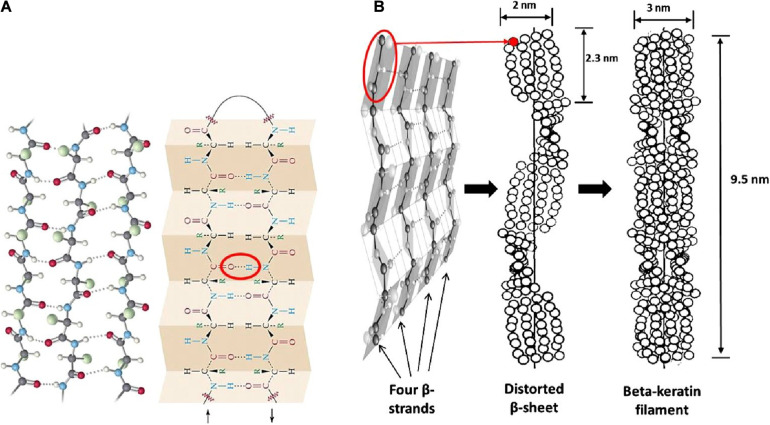
Structural representation of β-keratin filament; **(A)** ball and stick model of the protein chains, and β-pleated sheet consist of either parallel or antiparallel β-strands packed in a lateral manner, which also illustrate the intermolecular hydrogen bonds (red oval shape) that hold the polypeptides together; **(B)** schematic drawing of the formation of β-keratin; the folding of polypeptide chains at the central position gives four lateral β-strands, that associate with each other by the help of hydrogen bond to form a pleated sheet; and the polypeptides are also configured in a helical shape by distortion of the sheet; then, the superposition of the resultant pleated sheets that run in the opposite direction produces the β-keratin filament with a pitch length of 9.5 nm; reproduced from Wang B. et al. (2016) with permission (permission number: 4380240601835).

Epithelial tissues serving structural support functions possess keratin as one of the dominant family of fibrous proteins (Toivola et al., 2015). Keratin, a subtype of intermediate filament (IF) protein, is generally categorized into alpha (α) and beta (β) forms, showing characteristic structural assemblage with diameters of 7 and 3 nm, respectively (Figures 1B, 2B), buried in an amorphous matrix (Wang B. et al., 2016). The α- and β-forms have distinct synthetic pathways, which are reflected in their structures. Nonetheless, keratinocyte localized synthesis of both keratin forms involves coordinated and programmed sequential mechanisms with sets of gene activation and restriction processes (ul Haq and Akram, 2018). In vertebrates, keratins are encoded by 54 conserved genes and are controlled in pairs based on the type of tissues involved (Wang F. et al., 2016). The genes may be grouped into subtypes based on their evolutionary–functional relationship. The molecular architecture of keratin encoding genes in relation to other members of IFs suggests that their emergence was orchestrated by ancestral gene duplication during evolution (Wang F. et al., 2016). Keratinocytes are sites for keratin biosynthesis, and the synthetic processes are prudently coordinated by messenger ribonucleic acid (mRNA). Keratin formation is initiated by mitotic cell division accompany by the keratin proteins biosynthesis. Subsequently, the maturation of keratinocyte potentiates the arrest of cellular protein synthesis and also triggers nucleus degradation; these sequential activities prompt keratin stabilization and cell death (Wang B. et al., 2016). Keratins perform a series of functions in the epithelial tissues, including structural and non-structural cellular functions. However, these properties are conferred during posttranslational modifications, including phosphorylation and glycosylation (Lange et al., 2016), and also by some keratin-linked proteins (Bragulla and Homberger, 2009). Keratins also possess the tripartite structure uniformly shared among the IF proteins: “a highly conserved central α-helical rod domain flanked by a non-α-helical amino-terminal head domain and carboxy-terminal tail domain” (Nafeey et al., 2016). The unique pliable nature of IFs forms the basis for their role as a mechanical cushion that shields cells from external stressors (Leitner et al., 2012; Feroz et al., 2020). Additionally, keratin may be formed by the aggregation of coiled heterodimers consisting of an acidic and a basic subunit that are organized in an antiparallel manner. The two dimers stagger side by side to form a tetramer, and the tetramers associate in a lateral orientation to constitute a 10 nm diameter filament with a smooth surface and apolar characteristics and approximately made up of a cross-section of 16 coiled coils dimers (Wang F. et al., 2016). The variation in the molecular architecture and synthesis of the filament proteins are the important properties that distinguish α- and β-keratins (Jones et al., 1997), and these are represented in Figures 1, 2.

## Keratin Biodegradation – the Keratinase and Disulfide Reductase Synergy

**FIGURE 3:**
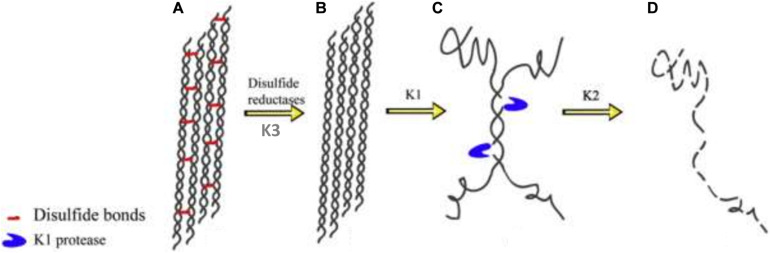
Degradation pathway of α-keratin by the keratinolytic proteases; **(A)** modeled structure of wool fibrils showing α-helix with densely cross-linked disulfide bonds; **(B)** wool fibrils already acted upon by disulfide reductase; **(C)** the keratinolytic protease (K1) unwinds the helices and cleaves the wool fibrils at strategic positions thereby exposing the peptide bonds to proteolytic attack; **(D)** K2 degrades the fibrils completely into their respective peptides and amino acids. The schematics were reproduced from Fang et al. (2013) with permission (permission number: 4206010207227).

Keratin mechanical durability is sustained by the inherent disulfide cross-linkages; therefore reduction of the densely populated disulfide bonds (sulfitolysis) in the keratin polypeptide enhances proteolytic cleavage, thus leading to enhanced degradation. As such, the detection of a high amount of thiol groups in the fermentation medium suggests a significant reduction of cysteine disulfide linkages within the α-helix motifs, which exposes the peptide bonds to proteolysis (Nnolim et al., 2020b). Consequently, the complete degradation of keratin may be achieved by the synergistic action of keratinolytic protease and disulfide bond reductase-like protein. There has been an extensive discussion on the mechanisms that bring about complete keratin degradation, which involve the actions of keratinase, disulfide reductase, sulfite or thiosulfate, and redox system (Brandelli et al., 2010). Scanning electron microscopy showed the effective combinatorial action of keratinolytic protease and disulfide bond reductase from *Bacillus* sp. on the structural perturbation of native keratin substrate compare to the separate activity of the keratinolytic protease supplemented with reducing agent or proteinase K (Rahayu et al., 2012). Yamamura et al. (2002) characterized two enzymes (keratinase and disulfide bond reductase) from *Stenotrophomonas* sp. D-1 that completely disintegrated keratin. Similarly, four extracellular keratinases with varying molecular weights, as confirmed by native PAGE zymography, from *Pseudomonas stutzeri* K4 showed cooperative actions toward the disintegration of keratin (Chaturvedi et al., 2014). Furthermore, Fang et al. (2013) showed the combined actions of three keratinolytic proteases designated as K1, K2, and K3 from *S. maltophilia* BB11-1 for keratin hydrolysis after their separation by chromatography. The respective participation of the enzymes in the keratinolytic process revealed K1 to have keratinase properties and to be mainly associated with keratin degradation (Figure 3); K2 possessed peptide bond hydrolysis properties, and K3 was a disulfide reductase involved in sulfitolysis.

The media nitrogen and carbon quotient influence microbial protease secretion and, keratinous proteins are inassimilable to microbial cells due to their structural complexity; therefore, production of elaborate keratinolytic enzymes may be regulated in response to media constituents (Riffel et al., 2011; Nnolim et al., 2020a). Worthy of note is the fact that several reports attributed unitary secreted enzymes to the complete degradation of keratin, thus controverting the position of cooperative enzyme actions. A comprehensive understanding of the mechanism behind keratinolysis still requires further studies, and the nature of individual keratinase should be put into perspective when defining the activity and potential application of the enzyme mixture.

## Keratinase Catalytic Efficiency Enhancement – a Protein Engineering Approach

Limitations associated with proteases derived from wild-type microbial species in industrial applications include low tolerance to some industrial operations and processing conditions such as pH, temperature, presence of organic solvents, salt, detergent, and oxidative agents. These factors drastically diminish the catalytic performance and applicability of the enzymes (Gupta and Ramnani, 2006; Brandelli et al., 2010; Hassan et al., 2020a). Keratinase, on the other hand, displays unique attributes that may be exploited in bioprocessing such as keratin bioconversion to useful products, textile treatment, leather depilation, and other industrial applications (Fang et al., 2013; Kalaikumari et al., 2019). Direct evolution (DE), site-directed mutagenesis (SDM), site-saturation mutagenesis (SSM), truncation, and fusion are among the protein engineering techniques that have been adopted to create robust enzymes (Gong et al., 2015; Yang et al., 2017; Vidmar and Vodovnik, 2018), and it has been successfully applied to overcome the limitations faced with biocatalysts (Fang et al., 2019). Site-directed mutagenesis depends on the protein structure alterations, meanwhile directed evolution functions based on a randomly generated library of mutants for the comprehensive discovery of promising variants (Gupta et al., 2013; Fang et al., 2017b). The protein alterations using SDM are achieved through gene modification by either targeted base(s) insertions, deletions, or substitutions. This protein engineering strategy changes the architecture of an enzyme, modulates the functional properties and catalytic mechanism (Yang et al., 2017). The protein residues at different locations, including non-catalytic residues, catalytic residues, the side chain residues, and residues in the catalytic triad vicinity, have been selectively engineered to obtain the desired biocatalyst (Takagi et al., 1988; Ashraf et al., 2019). Unlike SDM, DE involves non-specific/random mutagenesis to generate a library of variable genes, which are further screened for the selection of the best performing variants. This protein engineering strategy permits the enhancement and integration of much desirable potential into a single variant protein (Zhao and Feng, 2018). SSM is another unique laboratory approach to creating robust proteins, and it involves the targeted substitution of amino acid of an enzyme, possibly with any other naturally occurring amino acids (Siloto and Weselake, 2012). This approach produces clones that possess variable codons at the targeted site. Additionally, truncation entails removing some protein domains that are unnecessary for biocatalysis to enhance functionality. At the same time, fusion involves fusing vital domains from different enzymes to obtain a more catalytically active chimeric enzyme (Yang et al., 2017). With the foreknowledge that keratinolytic proteases belong to the subtilisin family, with high sequences homology, the various protein engineering strategies offer opportunities for modifying the biochemical properties as well as fine-tuning the functions of the enzymes.

### Modification for Improved Catalytic Efficiency

A study based on recombinant strains with a higher enzyme functionality using chemical mutagen gave a modified KD-N2 with enzyme activity that was 2.5 folds higher than the unmodified strain (Cai et al., 2008). A combination of ultraviolet radiation and sodium nitrite solution was used to create an efficient mutant strain of *B. subtilis* with 75% improvement on keratinase activity compare to the wild type (Zhang, 2012). Fang et al. (2017b) used a rational approach to improve on the catalysis of keratinase FDD and keratinase DDF by employing a mutagenesis-based approach and C-terminus maneuverability, respectively. Different substrate binding sites exist on the surface of an enzyme, and the cleft adjacent to the three catalytic residues (His, Asp, Ser) is known as S1 pocket, which is found in keratinases. The alterations of enzyme catalytic behavior coincide with the geometrical manipulations in the S1 cleft (Yan et al., 2009). The PPC domain is dominated by the single α-helix arranged opposite to the β-sheet to yield β-strands. Instances of sequence modification for improved catalytic activity include deletion of the C-terminal domain of the mutant MCP-03, which indicated that the C-terminus contributed less for the extracellular elaboration of the enzyme but may support enzyme function (Yan et al., 2009). Truncation of the C-terminal of HP70 significantly enhanced the enzyme activity with approximately fourfolds of the specific activity of SUC-AAPF-pNA (Ribitsch et al., 2010). The wild type keratinase (KerSMD) and the variants (without C-terminus) presented variable enzymatic activities on macromolecular and micromolecular substrates (Fang et al., 2016). Both the wild and variant KerSMD displayed significant proteolysis of soluble and insoluble proteinaceous substrates, suggesting that proper protein folding occurred regardless of whether either partial or complete C-terminal truncation was implemented. Therefore, truncation of C-terminus to optimize enzyme activity is promising, and its major function has been established to be associated with enhanced substrate recognition (Fang et al., 2016, 2017b), improved binding site affinity, and catalytic cleft enlargement (Fang et al., 2019).

### Modification to Switch Substrate Specificity

Keratinases producing microbes are limited, and the enzymes show a wide spectral distribution of molecular weight. Keratinases with relatively low molecular weight may solely have the catalytic part, while the medium to high molecular weight keratinases contain multi-domain structural assemblies that determine their substrate specificities (Kim et al., 2004). A conserved Gly166, situated beneath the active site of subtilisin BPN, was interchanged with the non-ionic residue to alter its specificity to other hydrophobic substrates (Estell et al., 1986). Wells et al. (1987) reported the exchange of substrate specificity of one enzyme for another by the maneuverability of the protein sequences at key positions. The maneuvering of the protein structure creates functionally divergent proteins, by the alteration of amino acid sequence, through protein engineering methods (Wells et al., 1987). These accounts are an indication of the feasibility to swap the substrate specificity of keratinase by altering the nature or the electrostatic affinity of the substrate-binding cleft with site-directed mutagenesis (Fang et al., 2015).

Collagen-degrading keratinase has limited application in the bioprocessing of leather products. However, substrate specificity alteration by protein engineering may pave the way for their potential application in the leather industry. Previously, it was reported that PPC domain of KerSMD presented sequence homology with vEP C-ter 100 that has a high affinity for collagen substrate (Fang et al., 2014). KerSMD was the most conspicuous extracellularly secreted keratinase of the pathogen *S. maltophilia* (Fang et al., 2014), which effectively degraded collagen and caused the laceration. The deletion of the C-terminus might circumvent collagen erosion by KerSMD, thereby broadening its potential candidacy in leather processing. Since the ideal protease employ in the bioprocessing of leather material needs keratinase activity for dehairing, but not collagenase activity; hence, alteration of substrate recognition of some collagen degrading keratinases may be justifiable and also beneficial from the industrial perspective.

### Modification for Chemical and pH Tolerance

Protease inclusion in modern laundry detergent as a major additive is essential for enhanced cleaning performance (Vojcic et al., 2015; Hammami et al., 2018). Keratinases are increasingly replacing the classic proteases in laundry detergent formulation because of their ability to hydrolyze an array of proteinaceous compounds and their relative stability with surfactants and organic solvents that are utilized in the detergent formulation (Paul et al., 2014c; Reddy et al., 2017). The impact of some chemical agents such as chaotropic agents and surfactants on protein structure may be an important parameter for assessment of the commercial value of a protein (Li et al., 2014). The C-terminus truncation greatly enhanced the tolerance of the keratinase from *S. maltophilia* to detergents and improved its enzymatic activity (Fang et al., 2016). A similar result was also achieved with a surfactant-stable keratinase from *S. aureofaciens* K13 (Gong et al., 2015). In addition, combined protein engineering of subtilisin E yielded robust mutant with about 40–60% residual enzyme activity in 4% (w/v) SDS (Li et al., 2014).

The PPC domain removal from V355 of KerSMD promoted the alkalinity – a 40% enhancement of enzyme activity was observed at pH 12 (Fang et al., 2016). The increase of negatively charged amino acids on the surface of a protein may have improved the stability of the enzyme under strong basic conditions (Graziano and Merlino, 2014). The processes of keratin biodegradation and leather processing are predominantly propagated in basic conditions that range from pH 8.0–12.0 (Fang et al., 2016); therefore, improving alkaline tolerance of keratinolytic enzymes becomes imperative for their optimal exploitations in eco-friendly developments.

### Modification for Halophilicity and Thermostability

Halo-tolerance is an important property of industrial enzymes (Yan et al., 2009; Takenaka et al., 2015). Halophilicity may be attributed to the ability of the protein amino acid residues to resist the destabilizing effect of surrounding electrolytes, and most of the halo-tolerant proteins have been described to have similar sequences dominated by acidic residues (Graziano and Merlino, 2014). The mutant KerSMDs retained significant enzymatic activity when compared to the wild type keratinase at a relatively high concentration of NaCl (15%; w/v) (Fang et al., 2016). Therefore, C-terminal truncation may be a good measure to determine the distribution of negatively charged residues on the protein surface, thereby enhancing halophilicity. The PPC domain of proteases also plays key role in thermostability of enzymes. Variant V370 with C-terminus total truncation was highly active at 60°C with enzyme activity of 5760 U/mg compared to the wild type counterpart (3280 U/mg) (Fang et al., 2016). Likewise, swapping of pro-sequences between *B. licheniformis* and *B. pumilus* keratinases yielded constructs with remarkable thermostability and extended half life time (Rajput et al., 2012). Additionally, complete deletion of MCP-03 C-terminus lowered the thermostability, suggesting its role in sustaining the catalytic integrity of the enzyme at a higher temperature (Yan et al., 2009).

## Environmental Impact of Keratinous Waste and the Ecological Implications of Keratinase Producing Microbes

The exponential increase in the global population is associated with pressure on agro-industries to increase production toward meeting the demand of the teeming population. Consequently, there is an increased generation of agro-industrial wastes, including keratinous wastes from the poultry processing farms, leather industry, and slaughterhouse (Tesfaye et al., 2017; Srivastava et al., 2020). Efficient management of the by-products, through recycling into value-added products and proper disposal of unusable waste, is of vital importance. In some instances, associated costs in the waste disposal process may be high if the known efficient means are applied to avert the detrimental effects of these wastes on the environment (Jaouadi et al., 2009). Lack of waste management regulatory measures and the non-adherence to the regulatory standard, where it exists, leave the environment littered with keratinous wastes, which in turn culminate in environmental pollution (Verma et al., 2017; Choińska-Pulit et al., 2019).

For instance, huge amount of hairs generated during leather processing as by-products are mostly landfilled or composted (Thankaswamy et al., 2018); with high tendency of exuding noxious gases that instigate air pollution due to the load of chemical constituents and microbial activity on the nitrogen-rich biomass (Akhter et al., 2020; Moktadir et al., 2020). Also, the leachate may contaminate the soil and groundwater, which would invariably constitute environmental concern and health risk to the local population (Ammasi et al., 2019). Similarly, discharging improperly treated or untreated wastewater with a high content of hair fragments in the water bodies would pose a threat to the aquatic life, as the tendency for eutrophication would be high.

The low economic importance and high mechanical stability of feathers result in their indiscriminate disposal and accumulation in the environment (Bagewadi et al., 2018). Most poultry farmers adopt the traditional methods of landfilling and incineration for the management of avian feathers. Keratinous feathers, because of their intractable nature, would persist for a long period of time in the environment; consequently, promoting the growth of variable microbial strains and also producing offensive odors due to the emission of air pollutants such as ammonia, nitrous oxide, and hydrogen sulfide (Patinvoh et al., 2016; Tamreihao et al., 2019). Likewise, incineration would significantly contribute to the greenhouse effect and other environmental problems (Cheong et al., 2018). The environmental pollution due to keratinous waste has the potential of becoming a global menace because the continuous growth and expansion of poultry and leather industries are imperative for providing affordable dietary protein and leather products, respectively (Tian et al., 2019; Emran et al., 2020).

The myriad of attributes for which microbes are endowed with play a significant role in the bio-geo-chemical processes in the environment. The maintenance of homeostatic balance in extreme situations, utilization of all known material (macro and micro), and elements as sources of energy and, or cellular building tools, are attributable to the diversity of the genome and the complexity of the microbial expression systems (Francino, 2012). Keratinases, are inducible enzymes that allow the producer microbe to utilize the keratinous biomass as a nutrient source (Gupta and Ramnani, 2006; Nnolim et al., 2020a), and the process occurs only in the presence of keratinous biomass and the absence of readily available nutritional reference; hence, keratinase production only kick-starts out of necessity. The exudation of keratinase offers microbes the opportunity to utilize a broad spectrum of proteinaceous substrates, thus promoting organic matter recycling in all ecological milieu (Bohacz and Korniłłowicz-Kowalska, 2019). The benefit conferred by keratinase producers to the environment includes the provision of assimilable nutrients through the dismemberment of keratinous biomass into bioavailable and utilizable units (Bohacz et al., 2020). The bio-decomposition process significantly promotes soil fertility via the supply, slow but regular release of organic nitrogenous compounds (Nafady et al., 2018). The foreseeable implication of the bioconversion of keratinous biomass into utilizable units include food chain nourishment, bio-geo-ecological balance, and safety to public and animal health as pathogens would have lost habitation consequent to organic matter decomposition (Kowalczyk et al., 2018; De Oliveira Martinez et al., 2020). Another significant contribution of the keratinase producing microbes is the recycling of refractory materials with keratin composition, thus, reducing the pollution levels of the planet (Callegaro et al., 2018).

Conversely, indiscriminate damage to proteinaceous component of cells and tissues by exuded keratinase may constitute another form of bio-hazard if it occurs in an unprecedented manner. However, limited manifestations have been reported on some pathogenic fungi that inhabit skin, causing mycosis and fur or hair grass-roots removal; and some dermatophytes including Arthroderma, Microsporum, Trichophyton, and Chrysosporium group have been in the fore (Huang et al., 2015; Bohacz et al., 2020).

## Application of Microbial Keratinolytic Proteases

**FIGURE 4:**
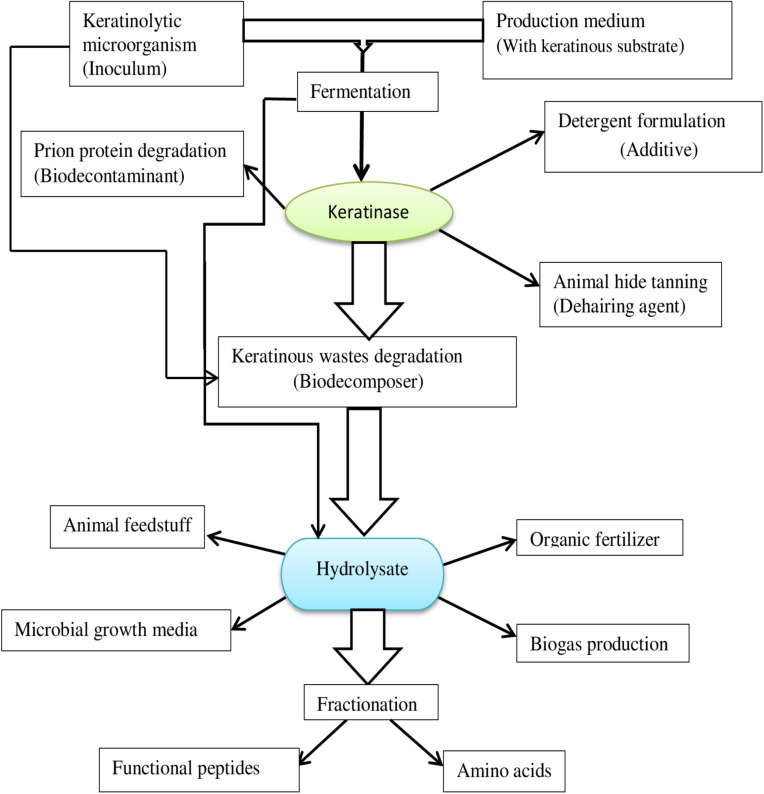
A flow chart of microbial keratinase application potential.

Keratinolytic proteases are incrementally gaining traction in industrial processes as an alternative to the classic chemical agents currently used. The invaluable potentials embedded in microbial keratinases are continuously evolving and are exploited in different bioprocesses (Figure 4), including but not limited to feed production, organic fertilizer production, detergent formulation, leather production, cosmetics, as well as in medicine and nanotechnology.

### Livestock Feed

The use of feather meal in supplementation of livestock feed has been in practice for decades; however, there has been concern about the nutritive value of the meal due to the unavailability of the utilizable protein in the feed. Keratins proteins in feathers and other keratinous resources are mostly inaccessible if the structural orientation contributed by different chemical groups is not significantly dismembered, because they are not digestible by ruminants or any other livestock (Williams et al., 1991; Grazziotin et al., 2008). Pressurized cooking or chemical treatment of feathers was the fundamental method of processing used in preparing feather meal. This method of processing involves high energy input, and it, therefore, denatures important heat-labile proteins (Onifade et al., 1998; Dong et al., 2017). The potentials presented by keratinolytic microbes and keratinases are an indication of the possibilities that the bio-recycling process holds for the husbandry sector of the agro-industry (Gegeckas et al., 2018). For instance, feed augmentation with *B. licheniformis* PWD-1 hydrolyzate, rich in free amino acids, led to growth improvement in chickens similar to the effect reported in those fed with soybean meal (Williams et al., 1991). Similarly, evaluation of feather hydrolyzate obtained using keratinase from *B. licheniformis* LMUB05 on birds performance showed that there was no significant difference between the broiler chicks on dietary inclusion and those fed with standard feed (Adetunji and Adejumo, 2018). Again, *B. licheniformis* ER-15 with high keratinolytic potentials produced extracellular dimeric keratinases with enhanced feather decomposition activity; decomposition occurred within 8 h of incubation at 50°C and pH 8, and the degree of feather hydrolysis was proportional to the amount of keratinase produced (Tiwary and Gupta, 2012). Considering the pressure on conventional feedstuff, e.g., soybean meal, which also serves as human food, and their high production cost, nutrient-rich hydrolyzates from keratinous wastes biodegradation are promising for use as an alternative in the formulation of affordable and adequate diet in animal production, due to their high protein contents and antioxidant potentials (Fakhfakh et al., 2011; Callegaro et al., 2018).

### Leather Production

Leather production is one of the highest-ranked socio-economic activities for many countries in the world. Because of the routine use of relatively toxic chemicals in leather processing, there has been a lot of health risk and ecological challenges associated with this practice (Hammami et al., 2018). Processing of animal hides entails various steps from the skin to the processed leather, which involves soaking, dehairing, bating, and tanning (Hamiche et al., 2019). Agents that could digest the protein-rich matter in the hides or skins are also applied in these rigorous operations, and one of the most polluting processes in leather making operations is the dehairing step (Adigüzel et al., 2009; Fang et al., 2017a). The classical method of animal skin dehairing uses lime-sulfide, which contributes significantly to the harmful nature of the final effluents, thus posing disposal problems (Kalaikumari et al., 2019). This operational procedure causes destruction of hair and also elevates loads of chemical oxygen demand (COD), biological oxygen demand (BOD), and total suspended solids (TSS) in the final effluent (Thankaswamy et al., 2018). The quest for cleaner technologies in the leather industry necessitates the search for eco-friendly dehairing approaches. Dehairing process with a proteolytic enzyme has been proposed as the convenient alternative, and keratinase has effectively displayed this desired quality (Tian et al., 2019). Some proteases damage collagen and give unacceptable physical properties to the finished leather (Macedo et al., 2005); therefore, keratinases that lack collagenolytic activity but possess mild elastinolytic activity have been increasingly employed as a suitable candidate for dehairing processes (Gong et al., 2015). Keratinases selectively degrade keratinous component of the follicle, hence, removing the intact hairs successfully without compromising the integrity of the leather material. Keratinolytic protease from *B. subtilis* S14 exhibiting excellent dehairing capabilities was proposed as a potential substitute for toxic sulfide in tanneries (Macedo et al., 2005). Keratinase-based dehairing being a “hair-saving dehairing” process, permits segregation of hair and reduces the large colloidal mixture and increased level of organic matter in effluents caused by the sulfide treatment process. A typical instance of the dehairing prospects of keratinases was reported by Jaouadi et al. (2013) where the enzyme (KERUS) was applied, and an exceptional dehairing activity on the sheep, goat, rabbit, and bovine skins was achieved. The dehairing processes are carried out under a relatively alkaline environment due to the nature of the sulfide and related chemical agents (Dayanandan et al., 2003); this matches the alkaline condition needed for optimum protease activity. The keratinase from *Vibrio* sp. Kr2 also attained a similar dehairing result but at a different pH range (6–8) and temperature (30°C) (Grazziotin et al., 2007). Another keratinase from *B. parabrevis* achieved complete removal of hair from goat hide after 7 h of incubation at 37°? and 220 rpm (Zhang et al., 2016). Microscopy of the enzymatically dehaired skin using SEM showed an intact skin with clearer hair pores. The lack of a collagenase activity by *B. parabrevis* keratinase suggested its candidacy for green processing of leather materials. The application of keratinase to animal skin processing in the leather industry has not only reduced the potential danger posed by the classical processes to the eco-system but has also provided the practices that can save energy globally (Jaouadi et al., 2013).

### Detergent Formulation

In contemporary times, using detergents formulated from bio-additives is preferred over the typical synthetic-based detergents. This is attributed to their effective cleaning attributes such as low-temperature washing compatibility, recalcitrant dirt removal properties, cloth fiber friendliness, and biodegradability (Jaouadi et al., 2009). The general performance of proteolytic enzymes in detergent is determined by several factors, including the washing medium pH, temperature, and component of the detergent (Reddy et al., 2017; Hammami et al., 2018). Ideally, proteases that are applied in the detergent making process must possess excellent activities and remain sufficiently stable within an increased spectrum of pH and temperatures, and also show a high degree of compatibility with other detergent ingredients, such as oxidizing and sequestering agents (Kumar and Takagi, 1999). Previously, the predominantly used detergent proteases in commercial products included Subtilisin Carlsberg (SC), Subtilisin Novo, Alcalase, Esperase, and Savinase (Outtrup et al., 1995). These proteases showed some levels of stability at conditions of elevated temperature and pH. During the preparation of bleach-based detergent, some active constituents like peroxide agents (H_2_O_2_), optical fiber brighteners, non-ionic surfactants (Tween-80), and anionic surfactant (SDS) are included, with the proteases usually showing a high level of inconsistency in the presence of these agents. Hence, such properties compromise their involvement in the present-day detergent formulation (Paul et al., 2016; Reddy et al., 2017). Therefore, there is a need to discover microbial enzymes that can function in the stipulated harsh conditions.

Microbial keratinases, on the other hand, is not hampered by surface-active agents, making it a good candidate for the formulation of both the liquid and solid detergents (Benkiar et al., 2013). In addition to their cost-effective production, keratinases have the tendency to hydrolyze recalcitrant and soluble substrates and function at broad temperature and pH ranges (Gupta et al., 2013; Gong et al., 2015; Paul et al., 2016). The efficient cleaning performance of keratinase from *Bacillus tequilensis* hsTKB2 was evaluated on blood and egg yolk stained fabrics (Paul et al., 2014c). Interestingly, the detergent supplemented with hsTKB2 keratinase showed an improved cleaning tendency compared to detergent alone. Likewise, blood-stained apron washed with detergent-keratinase mixture showed better cleaning power than detergent alone as observed from the respective cleaned fabrics’ reflectance and transmission (Paul et al., 2014b). Also, scanning electron microscopy revealed that the integrity of the fabrics was not compromised in the presence of keratinase, as observed from the smooth surface consistency of the cloths. Therefore, microbial keratinases have shown promising properties for application as ideal detergent bioadditive.

### Organic Fertilizer and Cosmetic Production

Keratin-rich wastes are known to have little or no application. Nonetheless, bioconversion of these high content keratin wastes may be an attractive approach toward valorization into cost-effective and eco-friendly resources for potential use as a slow-releasing nitrogen source for soil amendment (Gupta and Ramnani, 2006). The functions of the green fertilizer emanating from this keratinase-mediated wastes mineralization would include activation of plant growth; improve water retention capacity of soil and promotion of soil microbial activity that also supports phosphate solubility (Vasileva-Tonkova et al., 2009; Vidmar and Vodovnik, 2018). Hydrolyzed feather biomass generated by keratinolytic enzyme cocktail from a consortium of keratinolytic bacteria remarkably enhanced the germination of Gram seeds when compared to the test control (Jadhav and Pathade, 2019). Keratin hydrolyzate also serves as a rich source of phytohormone precursor – tryptophan for the synthesis of indole-3-acetic acid (IAA) (Tamreihao et al., 2019). While similar studies have reported direct production of IAA during decomposition of feather biomass by keratinase producing bacterial species (Jeong et al., 2010; Verma et al., 2016).

In the cosmetic sector, keratinases have been included as an active constituent in the preparation of topical products used as hair removals (Adelere and Lateef, 2016). Keratinases have shown potential to stiffen dead skin layer (hyperkeratosis) significantly present at appendages; hence, it could replace the conventionally used salicylic acid in a sustainable manner (Gupta and Ramnani, 2006). Different protein sources have been used in the production of hydrolyzates viz: wheat protein, wool keratin, and collagen, which have found applications in the production of hair and skincare products (Kshetri et al., 2020). Generally, they give an enhanced feel, moisture, and preserve the natural integrity of the skin (Barba et al., 2008; Villa et al., 2013). Peptides obtained by keratinase-mediated keratin biodegradation are of low molecular weights (Stiborova et al., 2016). These peptides tend to penetrate the hair or nail cuticles and have been increasingly included in the formulation of cosmetic products like moisturizers and conditioners compared to hydrolyzates from other sources (Villa et al., 2013). Sanghvi et al. (2016) investigated the dehairing efficiency of keratinase from *B. subtilis* DP1. Consequently, a bio-based depilatory agent was formulated with excipients whose compatibility and non-reactivity of the functional groups were also confirmed (Sanghvi et al., 2016). Interestingly, the keratinase-based formulated cream was more effective than the chemical-based counterpart in hair removal (Sanghvi et al., 2016).

### Other Potential Keratinase Applications

Keratinases have been reported to effectively destroy infective prion protein, and this might be due to the common similarities in the architecture of prion and keratin, such as fibrous and β-sheet-dominated proteins (Xia et al., 2012). Keratinase PWD-1 effectively degraded diseased prion protein (Langeveld et al., 2003). Mitsuiki et al. (2006) reported absolute decomposition of highly infective prion protein in 3 min by novel alkaline keratinolytic protease (NAPase) at pH 11 and 60°C. A study by Okoroma et al. (2013) implicated keratinase from keratinolytic *B. licheniformis* N22 coupled with bio-surfactant in complete degradation of the prion protein that causes scrapie after 10 min at 65°C.

Eco-friendly waste management is at the core of environmental sustainability. Bioenergy is often produced from an array of agro-industrial wastes with significant saccharide contents and requires basic construction technology, making it a sustainable fuel from both the economic and ecological points of view. The production involves a consolidated process that is initiated by biomass depolymerization; hence keratinase action on fibrous and intractable keratinous residues provides a starter material for this process. Chicken feathers pretreatment with recombinant keratinase from *B. megaterium* was reported to enhance biogas production (Forgács et al., 2011). Similarly, whole broth containing biodegradation products directed by wide-type keratinolytic *Bacillus* sp. C4 yields 124% increase in methane production against the yield from untreated feather (Patinvoh et al., 2016). Moreover, feather hydrolyzates generated through extracellular keratinase activity of *Bacillus* sp. CL18 yielded higher methane during anaerobic co-digestion than an untreated feather (Schommer et al., 2020). The protein-rich products obtain from keratinolytic digestion have been reported to provide, in addition to some proportion saccharides, assimilable nitrogen sources to support microbial growth during methanation (Branska et al., 2020).

Keratinase may also find application in the treatment of industrial wastewater. On that note, keratinase from *Meiothermus taiwanensis* WR-220 immobilized on cheap support; modified bagasse cellulose efficiently decolorized molasses wastewater with percentage decolorization that ranged from 84.7 to 90.2% (Zhang et al., 2019). The melanoidins removal performance of the keratinase was superior to other commercial immobilized enzymes tested, and this would open a new vista for yet another sustainable application of keratinase (Zhang et al., 2019).

Bio-digested avian feathers have shown more consistent results during analysis of non-protein biomolecules, including corticosterone when compare to other mechanical methods that present some limitations and technical concerns (Romero and Fairhurst, 2016). Consequently, keratinase from *Bacillus* sp. C4 promoted the extraction of glucocorticoids from chicken feather biomass (Alba et al., 2019). The efficiency of the enzymatic method was further confirmed by conducting parallelism test and hormone recovery employing radioimmunoassay.

## Future Recommendation

This study presented comprehensive progress in the field of microbial keratinase research. Efforts have been made to expand the applicability of keratinase because of its multifaceted application potentials for the implementation of green technological processes. However, one of the bottlenecks facing keratinase research is low yield even though many researchers have explored different yield enhancement strategies, at laboratory scale, to upgrade extracellular keratinase production by wild microorganisms. Importantly, molecular optimization (turning off some unnecessary metabolic pathways that compete with keratinase gene upregulation) of keratinase production could be combined with fundamental physico-chemical conditions construction if industrial-scale availability of the enzyme is envisaged. The development of such robust microbial strain would imperatively potentiate volumetric keratinase productivity. Furthermore, various microbial enzymes have been identified during the bio-decomposition of keratinous biomass by keratinolytic microbes. However, little is known about the action of these non-keratinase metabolites (lipase, cellulase, etc.) in mechanistic keratin degradation. Therefore, research into investigating the role of these enzymes in the biodegradation of keratinous materials portends a viable approach worthy of exploitation, as it may hold some potentialities for keratin-rich agroindustrial wastes valorization via microbial enzyme cocktail strategy. Additionally, a few keratinase-based formulations prepared predominantly from *B. licheniformis*, useful for improving the nutritional values of livestock feeds, have been commercialized so far. The commercialization of more products of similar nature would guarantee cheap livestock production; also, their preparation using microbial consortium could provide better feedstuffs laced with bioavailable nutrients. Lastly, microbial keratinase, at bench scale, has shown immense potential as a sustainable substitute for chemical dehairing agents used in leather processing. However, no known product is available in the market for the green processing of hide and skins at an industrial scale. Therefore, if put into perspective, the formulation of efficient keratinase-based unhairing products for industrial processing of hides would significantly reduce the environmental pollution and health hazard instigated by the fundamentally used chemical counterparts.

## Conclusion

Proteases have remarkably gained recognition as green catalysts with many prospects, with potential applications in several biotechnological and industrial processes. Keratinases, a group of proteases that effectively disintegrate the recalcitrant and insoluble keratin, has been gaining attention due to their many benefits. Microbial keratinase-mediated degradation of keratin-rich substrates has potentiated the sustainable valorization and bio-cycling of agro-industrial wastes in cost-effective and eco-friendly ways. The discovery of keratinases has significantly changed the technological processes for processing complex materials; from classic approaches to more sustainable bio-based methods.

Future investigation is needed to elucidate the mechanisms of keratinolysis since the process can be catalyzed by a single keratinase or in synergy with other enzymes. The robustness of keratinases has made it a highly important enzyme that would play a vital role in the present and future dynamics of the bio-economy. Besides, advances in technology can be used to improve the properties of microbial keratinases, potentially leading to new functionalities and applications in the future.

## Author Contributions

NN and UN contributed to conceptualization. UN contributed to the resources and funding acquisition. NN contributed to data curation, software, formal analysis, writing – original draft. CU contributed to writing – review and editing. UN and AO contributed to supervision, writing – review, and editing. All authors read and approved its final version.

## Conflict of Interest

The authors declare that the research was conducted in the absence of any commercial or financial relationships that could be construed as a potential conflict of interest.

## References

[B1] Abdel-Fattah A. M. El-Gamal M. S. Ismail S. A. Emran M. A. Hashem A. M. (2018). Biodegradation of feather waste by keratinase produced from newly isolated *Bacillus licheniformis* ALW1. *J. Genet. Eng. Biotechnol.* 16 311–318. 10.1016/j.jgeb.2018.05.005 30733740 PMC6353909

[B2] Abdel-fattah A. M. Nashy E. S. H. Sabiel E. T. A. Hussien M. M. Attia A. S. (2015). Novel keratinolytic activity of *Cyberlindnera fabianii* Nrc3 aza as a plant growth promoting agent (PGPA). *Int. J. Appl. Sci. Biotechnol.* 3 609–618. 10.3126/ijasbt.v3i4.13683

[B3] Abirami S. Ragavi R. Antony V. S. (2020). Utilization of keratinolytic *Lichtheimia corymbifera* AS1 for degradation of cattle hoove–a slaughter house waste to use in plant growth. *Biointerface Res. Appl. Chem.* 10 6417–6426. 10.33263/briac105.64176426

[B4] Adelere I. A. Lateef A. (2016). Keratinases: emerging trends in production and applications as novel multifunctional biocatalysts. *Kuwait J. Sci.* 43 118–127.

[B5] Adetunji C. O. Adejumo I. O. (2018). Efficacy of crude and immobilizedenzymes from *Bacillus licheniformis* for production of biodegraded feather meal and their assessment on chickens. *Environ. Technol. Innov.* 11 116–124. 10.1016/j.eti.2018.05.002

[B6] Adigüzel A. C. Bitlisli B. O. Yasa I. Eriksen N. T. (2009). Sequential secretion of collagenolytic, elastolytic, and keratinolytic proteases in peptide limited cultures of two *Bacillus cereus* strains isolated from wool. *J. Appl. Microbiol.* 107 226–234. 10.1111/j.1365-2672.2009.04200.x 19302303

[B7] Akhter M. Wal Marzan L. Akter Y. Shimizu K. (2020). Microbial bioremediation of feather waste for keratinase production: an outstanding solution for leather dehairing in tanneries. *Microbiol. Insights* 13:1178636120913280. 10.1177/1178636120913280 32440139 PMC7227156

[B8] Alahyaribeik S. Sharifi S. D. Tabandeh F. Honarbakhsh S. Ghazanfari S. (2020). Bioconversion of chicken feather wastes by keratinolytic bacteria. *Process Saf. Environ Prot.* 135 171–178. 10.1016/j.psep.2020.01.014

[B9] Alba A. C. Strauch T. A. Keisler D. H. Wells K. D. Kesler D. C. (2019). Using a keratinase to degrade chicken feathers for improved extraction of glucocorticoids. *Gen. and Comp. Endocrinol.* 270 35–40. 10.1016/j.ygcen.2018.10.002 30291864

[B10] Ammasi R. Victor J. S. Chellan R. Chellappa M. (2019). Amino acid enriched proteinous wastes: Recovery and reuse in leather making. *Waste Biomass Valor.* 11 5793–5807. 10.1007/s12649-019-00912-6

[B11] Anbu P. Gopinath S. C. B. Hilda A. Annadurai G. (2005). Purification of keratinase from poultry farm isolate-*Scopulariopsis brevicaulis* and statistical optimization of enzyme activity. *Enzyme Microb. Technol.* 36 639–647. 10.1016/j.enzmictec.2004.07.019

[B12] Anbu P. Hilda A. Sur H. W. Hur B. K. Jayanthi S. (2008). Extracellular keratinase from *Trichophyton* sp. HA-2 isolated from feather dumping soil. *Int. Biodeterior. Biodegradation* 62 287–292. 10.1016/j.ibiod.2007.07.017

[B13] Arokiyaraj S. Varghese R. Ahmed B. A. Duraipandiyan V. Al-Dhabi N. A. (2019). Optimizing the fermentation conditions and enhanced production of keratinase from *Bacillus cereus* isolated from halophilic environment. *Saudi J. Biol. Sci.* 26 378–381. 10.1016/j.sjbs.2018.10.011 31485181 PMC6717132

[B14] Ashraf N. M. Krishnagopal A. Hussain A. Kastner D. Sayed A. M. M. Mok Y. K. (2019). Engineering of serine protease for improved thermostability and catalytic activity using rational design. *Int. J. Biol. Macromol.* 126 229–237. 10.1016/j.ijbiomac.2018.12.218 30590144

[B15] Bach E. Daroit D. J. Corrêa A. P. F. Brandelli A. (2011). Production and properties of keratinolytic proteases from three novel Gram-negative feather-degrading bacteria isolated from Brazilian soils. *Biodegradation* 22 1191–1201. 10.1007/s10532-011-9474-0 21526391

[B16] Bach E. Sant’Anna V. Daroit D. J. Corrêa A. P. F. Segalin J. Brandelli A. (2012). Production, one-step purification, and characterization of a keratinolytic protease from *Serratia marcescens* P3. *Process Biochem.* 47 2455–2462. 10.1016/j.procbio.2012.10.007

[B17] Bagewadi Z. K. Sikandar I. M. Harichandra Z. N. (2018). Response surface methodology based optimization of keratinase production from *Trichoderma harzianum* isolate HZN12 using chicken feather waste and its application in dehairing of hide. *J. Environ. Chem. Eng.* 6 4828–4839. 10.1016/j.jece.2018.07.007

[B18] Barba C. Méndez S. Roddick-Lanzilotta A. Kelly R. Parra J. L. Coderch L. (2008). Cosmetic effectiveness of topically applied hydrolysed keratin peptides and lipids derived from wool. *Skin Res. Technol.* 14 243–248. 10.1111/j.1600-0846.2007.00280.x 18412569

[B19] Barman N. C. Zohora F. T. Das K. C. Mowla M. G. Banu N. A. Salimullah M. (2017). Production, partial optimization and characterization of keratinase enzyme by *Arthrobacter* sp. NFH5 isolated from soil samples. *AMB Express* 7:181. 10.1186/s13568-017-0462-6 28936604 PMC5608654

[B20] Benkiar A. Nadia Z. J. Badis A. Rebzani F. Soraya B. T. Rekik H. (2013). Biochemical and molecular characterisation of a thermo-and detergent-stable alkaline serine keratinolytic protease from *Bacillus circulans* strain DZ100 for detergent formulations and feather-biodegradation process. *Int. Biodeterior. Biodegrad.* 83 129–138. 10.1016/j.ibiod.2013.05.014

[B21] Bernal C. Cairo J. Coello N. (2006). Purification and characterisation of a novel exocellular keratinase from *Kocuria rosea*. *Enzyme Microb. Technol.* 38 49–54. 10.1016/j.enzmictec.2005.02.021

[B22] Bhange K. Chaturvedi V. Bhatt R. (2016). Simultaneous production of detergent stable keratinolytic protease, amylase and biosurfactant by *Bacillus subtilis* PF1 using agro industrial waste. *Biotechnol. Rep.* 10 94–104. 10.1016/j.btre.2016.03.007 28352529 PMC5040875

[B23] Bockle B. Galunsky B. Mueller R. (1995). Characterisation of a keratinolytic serine proteinase from *Streptomyces pactum* DSM 40530. *Appl. Environ. Microbiol.* 61 3705–3710. 10.1128/aem.61.10.3705-3710.1995 7487006 PMC167669

[B24] Bohacz J. Korniłłowicz-Kowalska T. (2019). Fungal diversity and keratinolytic activity of fungi from lignocellulosic composts with chicken feathers. *Process Biochem.* 80 119–128. 10.1016/j.procbio.2019.02.012

[B25] Bohacz J. Korniłłowicz-Kowalska T. Kitowski I. Ciesielska A. (2020). Degradation of chicken feathers by *Aphanoascus keratinophilus* and *Chrysosporium tropicum* strains from pellets of predatory birds and its practical aspect. *Int. Biodeterior. Biodegrad.* 151:104968 10.1016/j.ibiod.2020.104968

[B26] Bose A. Pathan S. Pathak K. Keharia H. (2014). Keratinolytic protease production by *Bacillus amyloliquefaciens* 6B using feather meal as substrate and application of feather hydrolysate as organic nitrogen input for agricultural soil. *Waste Biomass Valor.* 5 595–605. 10.1007/s12649-013-9272-5

[B27] Bouacem K. Bouanane-Darenfed A. Jaouadi N. Z. Joseph M. Hacene H. Ollivier B. (2016). Novel serine keratinase from *Caldicoprobacter algeriensis* exhibiting outstanding hide dehairing abilities. *Int. J. Biol. Macromol.* 86 321–328. 10.1016/j.ijbiomac.2016.01.074 26812107

[B28] Bragulla H. H. Homberger D. G. (2009). Structure and functions of keratin proteins in simple, stratified, keratinized and cornified epithelia. *J. Anat.* 214 516–559. 10.1111/j.1469-7580.2009.01066.x 19422428 PMC2736122

[B29] Brandelli A. (2008). Bacterial keratinases: useful enzymes for bioprocessing agroindustrial wastes and beyond. *Food Bioprocess Technol.* 1 105–116. 10.1007/s11947-007-0025-y

[B30] Brandelli A. Daroit D. J. Riffel A. (2010). Biochemical features of microbial keratinases and their production and applications. *Appl. Microbiol. Biotechnol.* 85 1735–1750. 10.1007/s00253-009-2398-5 20039036

[B31] Branska B. Foøtová L. Dvoøáková M. Liu H. Patakova P. Zhang J. (2020). Chicken feather and wheat straw hydrolysate for direct utilization in biobutanol production. *Renew. Energy* 145 1941–1948. 10.1016/j.renene.2019.07.094

[B32] Bressollier P. Letourneau F. Urdaci M. Verneuil B. (1999). Purification and characterisation of a keratinolytic serine proteinase from *Streptomyces albidoflavus*. *Appl. Environ. Microbiol.* 65 2570–2576. 10.1128/aem.65.6.2570-2576.1999 10347045 PMC91380

[B33] Brouta F. Descamps F. Fett T. Losson B. Gerday C. Mignon B. (2001). Purification and characterization of a 43? 5 kDa keratinolytic metalloprotease from *Microsporum canis*. *Med. Mycol.* 39 269–275. 11446530 10.1080/mmy.39.3.269.275

[B34] Cai C. G. Lou B. G. Zheng X. D. (2008). Keratinase production and keratin degradation by a mutant strain of *Bacillus subtilis*. *J. Zhejiang Univ. Sci. B* 9 60–67. 10.1631/jzus.b061620 18196614 PMC2170470

[B35] Callegaro K. Welter N. Daroit D. J. (2018). Feathers as bioresource: microbial conversion into bioactive protein hydrolysates. *Process Biochem.* 75 1–9. 10.1016/j.procbio.2018.09.002

[B36] Chaturvedi V. Bhange K. Bhatt R. Verma P. (2014). Production of kertinases using chicken feathers as substrate by a novel multifunctional strain of *Pseudomonas stutzeri* and its dehairing application. *Biocat. Agric. Biotechnol.* 3 167–174. 10.1016/j.bcab.2013.08.005

[B37] Cheong C. W. Lee Y. S. Ahmad S. A. Ooi P. T. Phang L. Y. (2018). Chicken feather valorization by thermal alkaline pretreatment followed by enzymatic hydrolysis for protein-rich hydrolysate production. *Waste Manage.* 79 658–666. 10.1016/j.wasman.2018.08.029 30343798

[B38] Choińska-Pulit A. Łaba W. Rodziewicz A. (2019). Enhancement of pig bristles waste bioconversion by inoculum of keratinolytic bacteria during composting. *Waste Manage.* 84 269–276. 10.1016/j.wasman.2018.11.052 30691901

[B39] Daroit D. J. Brandelli A. (2014). A current assessment on the production of bacterial keratinases. *Crit. Rev. Biotechnol.* 34 372–384. 10.3109/07388551.2013.794768 23937252

[B40] Dayanandan A. Kanagaraj J. Sounderraj L. Govindaraju R. Rajkumar G. S. (2003). Application of an alkaline protease in leather processing: an ecofriendly approach. *J. Clean. Prod.* 11 533–536. 10.1016/S0959-6526(02)00056-2

[B41] De Oliveira Martinez J. P. Cai G. Nachtschatt M. Navone L. Zhang Z. Robins K. (2020). Challenges and opportunities in identifying and characterising keratinases for value-added peptide production. *Catalysts* 10:184 10.3390/catal10020184

[B42] Dong Y. Z. Chang W. S. Chen P. T. (2017). Characterisation and overexpression of a novel keratinase from *Bacillus polyfermenticus* B4 in recombinant *Bacillus subtilis*. *Bioresour. Bioprocess.* 4:47 10.1186/s40643-017-0177-1

[B43] Emran M. A. Ismail S. A. Abdel-Fattah A. M. (2020). Valorization of feather via the microbial production of multi-applicable keratinolytic enzyme. *Biocatal. Agric. Biotechnol.* 27:101674 10.1016/j.bcab.2020.101674

[B44] Estell D. A. Graycar T. P. Miller J. V. Powers D. B. Burnier J. P. Ng P. G. (1986). Probing steric and hydrophobic effects on enzyme-substrate interactions by protein engineering. *Science* 233 659–664. 10.1126/science.233.4764.659 17835820

[B45] Fakhfakh N. Ktari N. Haddar A. Mnif I. H. Dahmen I. Nasri M. (2011). Total solubilisation of the chicken feathers by fermentation with a keratinolytic bacterium, *Bacillus pumilus* A1, and the production of protein hydrolysate with high antioxidative activity. *Process Biochem.* 46 1731–1737. 10.1016/j.procbio.2011.05.023

[B46] Falco F. C. Espersen R. Svensson B. Gernaey K. V. Lantz A. E. (2019). An integrated strategy for the effective production of bristle protein hydrolysate by the keratinolytic filamentous bacterium *Amycolatopsis keratiniphila* D2. *Waste Manage.* 89 94–102. 10.1016/j.wasman.2019.03.067 31079763

[B47] Fang Z. Sha C. Peng Z. Zhang J. Du G. (2019). Protein engineering to enhance keratinolytic protease activity and excretion in *Escherichia coli* and its scale-up fermentation for high extracellular yield. *Enzyme Microb. Technol.* 121 37–44. 10.1016/j.enzmictec.2018.11.003 30554643

[B48] Fang Z. Zhang J. Du G. Chen J. (2016). Improved catalytic efficiency, thermophilicity, anti-salt and detergent tolerance of keratinase KerSMD by partially truncation of PPC domain. *Sci. Rep.* 6:27953. 10.1038/srep27953 27298079 PMC4906391

[B49] Fang Z. Yong Y. C. Zhang J. Du G. Chen J. (2017a). Keratinolytic protease: a green biocatalyst for leather industry. *Appl. Microbiol. Biotechnol.* 101 7771–7779. 10.1007/s00253-017-8484-1 28924866

[B50] Fang Z. Zhang J. Du G. Chen J. (2017b). Rational protein engineering approaches to further improve the keratinolytic activity and thermostability of engineered keratinase KerSMD. *Biochem. Eng. J.* 127 147–153. 10.1016/j.bej.2017.08.010

[B51] Fang Z. Zhang J. Liu B. Du G. Chen J. (2013). Biochemical characterisation of three keratinolytic enzymes from *Stenotrophomonas maltophilia* BBE11-1 for biodegrading keratin wastes. *Int. Biodeterior. Biodegradation* 82 166–172. 10.1016/j.ibiod.2013.03.008

[B52] Fang Z. Zhang J. Liu B. Du G. Chen J. (2015). Insight into the substrate specificity of keratinase KerSMD from *Stenotrophomonas maltophilia* by site-directed mutagenesis studies in the S1 pocket. *RSC Adv.* 5 74953–74960. 10.1039/c5ra12598g

[B53] Fang Z. Zhang J. Liu B. Jiang L. Du G. Chen J. (2014). Cloning, heterologous expression and characterisation of two keratinases from *Stenotrophomonas maltophilia* BBE11-1. *Process Biochem.* 49 647–654. 10.1016/j.procbio.2014.01.009

[B54] Feroz S. Muhammad N. Ranayake J. Dias G. (2020). Keratin-Based materials for biomedical applications. *Bioact. Mater.* 5 496–509. 10.1016/j.bioactmat.2020.04.007 32322760 PMC7171262

[B55] Ferrareze P. A. G. Correa A. P. F. Brandelli A. (2016). Purification and characterization of a keratinolytic protease produced by probiotic *Bacillus subtilis*. *Biocatal. Agric. Biotechnol.* 7 102–109. 10.1016/j.bcab.2016.05.009

[B56] Forgács G. Alinezhad S. Mirabdollah A. Feuk-Lagerstedt E. Horváth I. S. (2011). Biological treatment of chicken feather waste for improved biogas production. *J. Environ. Sci.* 23 1747–1753. 10.1016/s1001-0742(10)60648-1 22432272

[B57] Francino M. P. (2012). The ecology of bacterial genes and the survival of the new. *Int. J. Evol. Biol.* 2012:394026. 10.1155/2012/394026 22900231 PMC3415099

[B58] Friedrich A. B. Antranikian G. (1996). Keratin degradation by *Fervidobacterium pennavorans*, a novel thermophilic anaerobic species of the order thermotogales. *Appl. Environ. Microbiol.* 62 2875–2882. 10.1128/aem.62.8.2875-2882.1996 16535379 PMC1388917

[B59] Gegeckas A. Šimkutë A. Gudiukaitë R. Èitavièius D. J. (2018). Characterisation and application of keratinolytic paptidases from *Bacillus* spp. *Int. J. Biol. Macromol.* 113 1206–1213. 10.1016/j.ijbiomac.2018.03.046 29545060

[B60] Gong J. S. Wang Y. Zhang D. D. Zhang R. X. Su C. Li H. (2015). Biochemical characterisation of an extreme alkaline and surfactant-stable keratinase derived from a newly isolated actinomycete *Streptomyces aureofaciens* K13. *RSC Adv.* 5 24691–24699. 10.1039/c4ra16423g

[B61] Gradišar H. Friedrich J. Križaj I. Jerala R. (2005). Similarities and specificities of fungal keratinolytic proteases: comparison of keratinases of *Paecilomyces marquandii* and *Doratomyces microsporus* to some known proteases. *Appl. Environ. Microbiol.* 71 3420–3426. 10.1128/aem.71.7.3420-3426.2005 16000744 PMC1168971

[B62] Gradišar H. Kern S. Friedrich J. (2000). Keratinase of *Doratomyces microsporus*. *Appl. Microbiol. Biotechnol.* 53 196–200. 10.1007/s002530050008 10709982

[B63] Graziano G. Merlino A. (2014). Molecular bases of protein halotolerance. *Biochim. Biophys. Acta Proteins Proteomics* 1844 850–858. 10.1016/j.bbapap.2014.02.018 24590113

[B64] Grazziotin A. Pimentel F. A. De Jong E. V. Brandelli A. (2008). Poultry feather hydrolysate as a protein source for growing rats. *Braz. J. Vet. Res. An. Sci.* 45 61–67.

[B65] Grazziotin A. Pimentel F. A. Sangali S. de Jong E. V. Brandelli A. (2007). Production of feather protein hydrolysate by keratinolytic bacterium *Vibrio* sp. kr2. *Bioresour. Technol.* 98 3172–3175. 10.1016/j.biortech.2006.10.034 17223559

[B66] Guo L. Lu L. Yin M. Yang R. Zhang Z. Zhao W. (2020). Valorization of refractory keratinous waste using a new and sustainable bio-catalysis. *Chem. Eng. J.* 397:125420 10.1016/j.cej.2020.125420

[B67] Gupta R. Ramnani P. (2006). Microbial keratinases and their prospective applications: an overview. *Appl. Microbiol. Biotechnol.* 70:21. 10.1007/s00253-005-0239-8 16391926

[B68] Gupta R. Sharma R. Beg Q. K. (2013). Revisiting microbial keratinases: next generation proteases for sustainable biotechnology. *Crit. Rev. Biotechnol.* 33 216–228. 10.3109/07388551.2012.685051 22642703

[B69] Habbeche A. Saoudi B. Jaouadi B. Haberra S. Kerouaz B. Boudelaa M. (2014). Purification and biochemical characterisation of a detergent-stable keratinase from a newly thermophilic actinomycete *Actinomadura keratinilytica* strain Cpt29 isolated from poultry compost. *J. Biosci. Bioeng.* 117 413–421. 24140106 10.1016/j.jbiosc.2013.09.006

[B70] Hamiche S. Mechri S. Khelouia L. Annane R. El Hattab M. Badis A. (2019). Purification and biochemical characterization of two keratinases from *Bacillus amyloliquefaciens* S13 isolated from marine brown alga Zonaria tournefortii with potential keratin-biodegradation and hide-unhairing activities. *Int. J. Biol. Macromol.* 122 758–769. 10.1016/j.ijbiomac.2018.10.174 30389529

[B71] Hammami A. Fakhfakh N. Abdelhedi O. Nasri M. Bayoudh A. (2018). Proteolytic and amylolytic enzymes from a newly isolated *Bacillus mojavensis* SA: characterization and applications as laundry detergent additive and in leather processing. *Int. J. Biol. Macromol.* 108 56–68. 10.1016/j.ijbiomac.2017.11.148 29180048

[B72] Hassan M. A. Abol-Fotouh D. Omer A. M. Tamer T. M. Abbas E. (2020a). Comprehensive insights into microbial keratinases and their implication in various biotechnological and industrial sectors: a review. *Int. J. Biol. Macromol.* 154 567–583. 10.1016/j.ijbiomac.2020.03.116 32194110

[B73] Hassan M. A. Taha T. H. Hamad G. M. Hashem M. Alamri S. Mostafa Y. S. (2020b). Biochemical characterisation and application of keratinase from *Bacillus thuringiensis* MT1 to enable valorisation of hair wastes through biosynthesis of vitamin B-complex. *Int. J. Biol. Macromol.* 153 561–572. 10.1016/j.ijbiomac.2020.03.032 32151720

[B74] Hossain M. S. Azad A. K. Sayem S. A. Mostafa G. Hoq M. M. (2007). Production and partial characterisation of feather-degrading keratinolytic serine protease from *Bacillus licheniformis* MZK-3. *J. Biol. Sci.* 7 599–606. 10.3923/jbs.2007.599.606

[B75] Huang Y. Busk P. K. Herbst F. A. Lange L. (2015). Genome and secretome analyses provide insights into keratin decomposition by novel proteases from the non-pathogenic fungus *Onygena corvina*. *Appl. Microbiol. Biotechnol.* 99 9635–9649. 10.1007/s00253-015-6805-9 26177915 PMC4628079

[B76] Jadhav P. Pathade G. (2019). Degradation of feathers by bacterial consortium and its application in seed germination. *Int. Res. J. Biol. Sci.* 8 20–23.

[B77] Jaouadi B. Abdelmalek B. Fodil D. Ferradji F. Z. Rekik H. Zaraî N. (2010). Purification and characterisation of a thermostable keratinolytic serine alkaline proteinase from *Streptomyces* sp. strain AB1 with high stability in organic solvents. *Bioresour. Technol.* 101 836–8369. 20624606 10.1016/j.biortech.2010.05.066

[B78] Jaouadi B. Ellouz-Chaabouni S. Ali M. B. Messaoud E. B. Naili B. Dhouib A. (2009). Excellent laundry detergent compatibility and high dehairing ability of the *Bacillus pumilus* CBS alkaline proteinase (SAPB). *Biotechnol. Bioprocess Eng.* 14 503–512. 10.1007/s12257-008-0244-8

[B79] Jaouadi B. Ellouz-Chaabouni S. Rhimi M. Bejar S. (2008). Biochemical and molecular characterisation of a detergent-stable serine alkaline protease from *Bacillus pumilus* CBS with high catalytic efficiency. *Biochimie* 90 1291–1305. 10.1016/j.biochi.2008.03.004 18397761

[B80] Jaouadi N. Z. Rekik H. Badis A. Trabelsi S. Belhoul M. Yahiaoui A. B. (2013). Biochemical and molecular characterisation of a serine keratinase from *Brevibacillus brevis* US575 with promising keratin-biodegradation and hide-dehairing activities. *PLoS One* 8:e76722. 10.1371/journal.pone.0076722 24146914 PMC3795758

[B81] Jeong J. H. Lee O. M. Jeon Y. D. Kim J. D. Lee N. R. Lee C. Y. (2010). Production of keratinolytic enzyme by a newly isolated feather-degrading *Stenotrophomonas maltophilia* that produces plant growth-promoting activity. *Process Biochem.* 45 1738–1745. 10.1016/j.procbio.2010.07.020

[B82] Jin M. Chen C. He X. Zeng R. (2019). Characterization of an extreme alkaline-stable keratinase from the draft genome of feather-degrading *Bacillus* sp. JM7 from deep-sea. *Acta Oceanol. Sin.* 38 87–95. 10.1007/s13131-019-1350-5

[B83] Jones L. N. Simon M. Watts N. R. Booy F. P. Steven A. C. Parry D. A. D. (1997). Intermediate filament structure: hard α-keratin. *Biophys. Chem.* 68 83–93. 10.1016/s0301-4622(97)00013-6 9468612

[B84] Kalaikumari S. S. Vennila T. Monika V. Chandraraj K. Gunasekaran P. Rajendhran J. (2019). Bioutilization of poultry feather for keratinase production and its application in leather industry. *J. Clean. Prod.* 208 44–53. 10.1016/j.jclepro.2018.10.076

[B85] Kang D. Herschend J. Al-Soud W. A. Mortensen M. S. Gonzalo M. Jacquiod S. (2018). Enrichment and characterization of an environmental microbial consortium displaying efficient keratinolytic activity. *Bioresour. Technol.* 270 303–310. 10.1016/j.biortech.2018.09.006 30236907

[B86] Kim J. D. (2007). Purification and characterisation of a keratinase from a feather-degrading fungus, *Aspergillus flavus* Strain K-03. *Mycobiology* 32 219–225. 10.4489/myco.2007.35.4.219 24015101 PMC3763176

[B87] Kim J. S. Kluskens L. D. de Vos W. M. Huber R. van der Oost J. (2004). Crystal structure of fervidolysin from *Fervidobacterium pennivorans*, a keratinolytic enzyme related to subtilisin. *J. Mol. Biol.* 335 787–797. 10.1016/j.jmb.2003.11.006 14687574

[B88] Kojima M. Kanai M. Tominaga M. Kitazume S. Inoue A. Horikoshi K. (2006). Isolation and characterisation of a feather-degrading enzyme from *Bacillus pseudofirmus* FA30-01. *Extremophiles* 10 229–235. 10.1007/s00792-005-0491-y 16489414

[B89] Korniłłowicz-Kowalska T. Bohacz J. (2011). Biodegradation of keratin waste: theory and practical aspects. *Waste Manage.* 31 1689–1701. 10.1016/j.wasman.2011.03.024 21550224

[B90] Kowalczyk P. Mahdi-Oraibi S. Misiewicz A. Gabzdyl N. Miskiewicz A. Szparecki G. (2018). Feather-degrading bacteria: their biochemical and genetic characteristics. *Arab. J. Sci. Eng.* 43 33–41. 10.1007/s13369-017-2700-2

[B91] Kshetri P. Roy S. S. Chanu S. B. Singh T. S. Tamreihao K. Sharma S. K. (2020). Valorization of chicken feather waste into bioactive keratin hydrolysate by a newly purified keratinase from *Bacillus* sp. RCM-SSR-102. *J. Environ. Manage.* 273:111195 10.1016/j.jenvman.2020.111195 32771848

[B92] Kumar C. G. Takagi H. (1999). Microbial alkaline proteases: from a bioindustrial viewpoint. *Biotechnol. Adv.* 17 561–594. 10.1016/s0734-9750(99)00027-0 14538129

[B93] Kunert J. (2000). Physiology of keratinophilic fungi. *Rev. Iberoam. Micol.* 1 77–85.

[B94] Kuo J. M. Yang J. I. Chen W. M. Pan M. H. Tsai M. L. Lai Y. J. (2012). Purification and characterisation of a thermostable keratinase from *Meiothermus* sp. I40. *Int. Biodeterior. Biodegradation* 70 111–116. 10.1016/j.ibiod.2012.02.006

[B95] Łaba W. Szczekała K. B. (2013). Keratinolytic proteases in biodegradation of pretreated feathers. *Pol. J. Environ. Stud.* 22 1101–1109.

[B96] Lange L. Huang Y. Busk P. K. (2016). Microbial decomposition of keratin in nature—a new hypothesis of industrial relevance. *Appl. Microbiol. Biotechnol.* 100 2083–2096. 10.1007/s00253-015-7262-1 26754820 PMC4756042

[B97] Langeveld J. P. Wang J. J. Van de Wiel D. F. Shih G. C. Garssen G. J. Bossers A. (2003). Enzymatic degradation of prion protein in brain stem from infected cattle and sheep. *J. Infect. Dis.* 188 1782–1789. 10.1086/379664 14639552

[B98] Lee Y. J. Dhanasingh I. Ahn J. S. Jin H. S. Choi J. M. Lee S. H. (2015). Biochemical and structural characterisation of a keratin-degrading M32 carboxypeptidase from *Fervidobacterium islandicum* AW-1. *Biochem. Biophys. Res. Commun.* 468 927–933. 10.1016/j.bbrc.2015.11.058 26603937

[B99] Leitner A. Paust T. Marti O. Walther P. Herrmann H. Beil M. (2012). Properties of intermediate filament networks assembled from keratin 8 and 18 in the presence of Mg2+. *Biophys. J.* 103 195–201. 10.1016/j.bpj.2012.06.014 22853896 PMC3403007

[B100] Letourneau F. Soussotte V. Bressollier P. Branland P. Verneuil B. (1998). Keratinolytic activity of *Streptomyces* sp. S. K1-02: a new isolated strain. *Lett. Appl. Microbiol.* 26 77–80. 10.1046/j.1472-765x.1998.00281.x 9489039

[B101] Li Q. (2019). Progress in microbial degradation of feather waste. *Front. Microbiol.* 10:2717. 10.3389/fmicb.2019.02717 31866957 PMC6906142

[B102] Li Z. Roccatano D. Lorenz M. Martinez R. Schwaneberg U. (2014). Insights on activity and stability of subtilisin E towards guanidinium chloride and sodium dodecylsulfate. *J. Biotechnol.* 169 87–94. 10.1016/j.jbiotec.2013.11.001 24280236

[B103] Lin H. H. Yin L. J. Jiang S. T. (2009). Expression and purification of *Pseudomonas aeruginosa* keratinase in *Bacillus subtilis* DB104 expression system. *J. Agric. Food Chem.* 57 7779–7784. 10.1021/jf901903p 19722707

[B104] Lv L. X. Sim M. H. Li Y. D. Min J. Feng W. H. Guan W. J. (2010). Production, characterization and application of a keratinase from *Chryseobacterium* L99 sp. nov. *Process Biochem.* 45 1236–1244. 10.1016/j.procbio.2010.03.011

[B105] Macedo A. J. da Silva W. O. B. Gava R. Driemeier D. Henriques J. A. P. Termignoni C. (2005). Novel keratinase from *Bacillus subtilis* S14 exhibiting remarkable dehairing capabilities. *Appl. Environ. Microbiol.* 71 594–596. 10.1128/aem.71.1.594-596.2005 15640244 PMC544270

[B106] Mitsuiki S. Hui Z. Matsumoto D. Sakai M. Moriyama Y. Furukawa K. (2006). Degradation of PrPSc by keratinolytic protease from *Nocardiopsis* sp. TOA-1. *Biosci. Biotechnol. Biochem.* 70 1246–1248. 10.1271/bbb.70.1246 16717429

[B107] Moktadir M. A. Ahmadi H. B. Sultana R. Liou J. J. Rezaei J. (2020). Circular economy practices in the leather industry: A practical step towards sustainable development. *J. Clean Prod.* 251:119737 10.1016/j.jclepro.2019.119737

[B108] Nafady N. A. Hassan E. A. Abd-Alla M. H. Bagy M. M. K. (2018). Effectiveness of eco-friendly arbuscular mycorrhizal fungi biofertilizer and bacterial feather hydrolysate in promoting growth of Vicia faba in sandy soil. *Biocatal. Agric. Biotechnol.* 16 140–147. 10.1016/j.bcab.2018.07.024

[B109] Nafeey S. Martin I. Felder T. Walther P. Felder E. (2016). Branching of keratin intermediate filaments. *J. Struct. Biol.* 194 415–422. 10.1016/j.jsb.2016.03.023 27039023

[B110] Nam G. W. Lee D. W. Lee H. S. Lee N. J. Kim B. C. Choe E. A. (2002). Native-feather degradation by *Fervidobacterium islandicum* AW-1, a newly isolated keratinase-producing thermophilic anaerobe. *Arch. Microbiol.* 178 538–547. 10.1007/s00203-002-0489-0 12420177

[B111] Navone L. Speight R. (2018). Understanding the dynamics of keratin weakening and hydrolysis by proteases. *PLoS One* 13:e0202608. 10.1371/journal.pone.0202608 30114294 PMC6095591

[B112] Nnolim N. E. Okoh A. I. Nwodo U. U. (2020a). *Bacillus* sp. fpf-1 produced keratinase with high potential for chicken feather degradation. *Molecules* 25:150 10.3390/molecules250715055 PMC7180861 32225031

[B113] Nnolim N. E. Okoh A. I. Nwodo U. U. (2020b). Proteolytic bacteria isolated from agro-waste dumpsites produced keratinolytic enzymes. *Biotechnol. Rep.* 27:e00483 10.1016/j.btre.2020.e00483 PMC7267708 32514407

[B114] Okoroma E. A. Purchase D. Garelick H. Morris R. Neale M. H. Windl O. (2013). Enzymatic formulation capable of degrading scrapie prion under mild digestion conditions. *PLoS One* 8:e68099. 10.1371/journal.pone.0068099 23874511 PMC3712960

[B115] Onifade A. A. Al-Sane N. A. Al-Musallam A. A. Al-Zarban S. (1998). A review: potentials for biotechnological applications of keratin-degrading microorganisms and their enzymes for nutritional improvement of feathers and other keratins as livestock feed resources. *Bioresour. Technol.* 66 1–11. 10.1016/s0960-8524(98)00033-9

[B116] Outtrup H. Dambmann C. Christiansen M. Aaslyng D. A. Nove Nordisk A. S. (1995). *Alkaline Protease Bacillus sp. JP 395, Method of Making and Detergent Compositions.* U.S. Patent 5466594 Brussels: EC.

[B117] Parra R. Aldred D. Magan N. (2005). Medium optimization for the production of the secondary metabolite squalestatin S1 by a *Phoma* sp. combining orthogonal design and response surface methodology. *Enzyme Microb. Technol.* 37 704–711. 10.1016/j.enzmictec.2005.04.009

[B118] Patinvoh R. J. Feuk-Lagerstedt E. Lundin M. Horváth I. S. Taherzadeh M. J. (2016). Biological pretreatment of chicken feather and biogas production from total broth. *Appl. Biochem. Biotechnol.* 180 1401–1415. 10.1007/s12010-016-2175-8 27350050

[B119] Paul T. Das A. Mandal A. Halder S. K. DasMohapatra P. K. Pati B. R. (2014a). Production and purification of keratinase using chicken feather bioconversion by a newly isolated *Aspergillus fumigatus* TKF1: detection of valuable metabolites. *Biomass Convers. Biorefin.* 4 137–148. 10.1007/s13399-013-0090-6

[B120] Paul T. Das A. Mandal A. Halder S. K. DasMohapatra P. K. Pati B. R. (2014b). Valorisation of chicken feather waste for production of keratinase, oligopeptides and essential amino acids under submerged fermentation by *Paenibacillus woosongensis* TKB2. *Waste Biomass Valor.* 5 575–584. 10.1007/s12649-013-9267-2

[B121] Paul T. Das A. Mandal A. Halder S. K. Jana A. Maity C. (2014c). An efficient cloth cleaning properties of a crude keratinase combined with detergent: towards industrial viewpoint. *J. Clean. Prod.* 66 672–684. 10.1016/j.jclepro.2013.10.054

[B122] Paul T. Jana A. Mandal A. K. Mandal A. Mohpatra P. K. D. Mondal K. C. (2016). Bacterial keratinolytic protease, imminent starter for NextGen leather and detergent industries. *Sustain. Chem. Pharm.* 3 8–22. 10.1016/j.scp.2016.01.001

[B123] Pereira J. Q. Lopes F. C. Petry M. V. da Costa Medina L. F. Brandelli A. (2014). Isolation of three novel Antarctic psychrotolerant feather-degrading bacteria and partial purification of keratinolytic enzyme from *Lysobacter* sp. A03. *Int. Biodeterior. Biodegrad.* 88 1–7. 10.1016/j.ibiod.2013.11.012

[B124] Rahayu S. Syah D. Suhartono M. T. (2012). Degradation of keratin by keratinase and disulfide reductase from *Bacillus* sp. MTS of Indonesian origin. *Biocat. Agric Biotechnol.* 1 152–158. 10.1016/j.bcab.2012.02.001

[B125] Rajput R. Tiwary E. Sharma R. Gupta R. (2012). Swapping of pro-sequences between keratinases of *Bacillus licheniformis* and *Bacillus pumilus*: altered substrate specificity and thermostability. *Enzyme Microb. Technol.* 51 131–138. 10.1016/j.enzmictec.2012.04.010 22759531

[B126] Ramnani P. Singh R. Gupta R. (2005). Keratinolytic potential of *Bacillus licheniformis* RG1: structural and biochemical mechanism of feather degradation. *Can. J. Microbiol.* 51 191–196. 10.1139/w04-123 15920616

[B127] Rawlings N. D. (2016). Peptidase specificity from the substrate cleavage collection in the MEROPS database and a tool to measure cleavage site conservation. *Biochimie* 122 5–30. 10.1016/j.biochi.2015.10.003 26455268 PMC4756867

[B128] Reddy M. R. Reddy K. S. Chouhan Y. R. Bee H. Reddy G. (2017). Effective feather degradation and keratinase production by *Bacillus pumilus* GRK for its application as bio-detergent additive. *Bioresour. Technol.* 243 254–263. 10.1016/j.biortech.2017.06.067 28672188

[B129] Ribitsch D. Karl W. Birner-Gruenberger R. Gruber K. Eiteljoerg I. Remler P. (2010). C-terminal truncation of a metagenome-derived detergent protease for effective expression in *E. coli*. *J. Biotechnol.* 150 408–416. 10.1016/j.jbiotec.2010.09.947 20869404

[B130] Rieger T. J. De Oliveira C. T. Pereira J. Q. Brandelli A. Daroit D. J. (2017). Proteolytic system of *Bacillus* sp. CL18 is capable of extensive feather degradation and hydrolysis of diverse protein substrates. *Br. Poult. Sci.* 58 329–335. 10.1080/00071668.2017.1293229 28277791

[B131] Riessen S. Antranikian G. (2001). Isolation of *Thermoanaerobacter* keratinophilus sp. nov., a novel thermophilic, anaerobic bacterium with keratinolytic activity. *Extremophiles* 5 399–408. 10.1007/s007920100209 11778841

[B132] Riffel A. Brandelli A. (2006). Keratinolytic bacteria isolated from feather waste. *Brazilian J. Microbiol.* 37 395–399. 10.1590/s1517-83822006000300036

[B133] Riffel A. Daroit D. J. Brandelli A. (2011). Nutritional regulation of protease production by the feather-degrading bacterium *Chryseobacterium* sp. kr6. *New Biotechnol.* 28 153–157. 10.1016/j.nbt.2010.09.008 20920618

[B134] Riffel A. Ortolan S. Brandelli A. (2003). De-hairing activity of extracellular proteases produced by keratinolytic bacteria. *J. Chem. Technol. Biotechnol.* 78 855–859. 10.1002/jctb.828

[B135] Romero L. M. Fairhurst G. D. (2016). Measuring corticosterone in feathers: strengths, limitations, and suggestions for the future. *Comp. Biochem. Physiol. Part A Mol. Integr. Physiol.* 202 112–122. 10.1016/j.cbpa.2016.05.002 27155053

[B136] Sangali S. Brandelli A. (2000). Feather keratin hydrolysis by a *Vibrio* sp. strain kr2. *J. Appl. Microbiol.* 89 735–743. 10.1046/j.1365-2672.2000.01173.x 11119146

[B137] Sanghvi G. Patel H. Vaishnav D. Oza T. Dave G. Kunjadia P. (2016). A novel alkaline keratinase from *Bacillus subtilis* DP1 with potential utility in cosmetic formulation. *Int. J. Biol. Macromol.* 87 256–262. 10.1016/j.ijbiomac.2016.02.067 26940376

[B138] Sapkota A. R. Lefferts L. Y. McKenzie S. Walker P. (2007). What do we feed to food-production animals? A review of animal feed ingredients and their potential impacts on human health. *Environ. Health Perspect.* 115 663–670. 10.1289/ehp.9760 17520050 PMC1867957

[B139] Schommer V. A. Wenzel B. M. Daroit D. J. (2020). Anaerobic co-digestion of swine manure and chicken feathers: Effects of manure maturation and microbial pretreatment of feathers on methane production. *Renew. Energy* 152 1284–1291. 10.1016/j.renene.2020.01.154

[B140] Schrooyen P. M. Dijkstra P. J. Oberthür R. C. Bantjes A. Feijen J. (2001). Partially carboxymethylated feather keratins. 2. Thermal and mechanical properties of films. *J. Agric. Food Chem.* 49 221–230. 10.1021/jf0004154 11170581

[B141] Selvam K. Vishnupriya B. (2012). Biochemical and molecular characterisation of microbial keratinase and its remarkable applications. *Int. J. Pharm. Biol. Arch.* 3 267–275.

[B142] Sharma M. Sharma M. Rao V. M. (2011). In vitro biodegradation of keratin by dermatophytes and some soil keratinophiles. *Afr. J. Biochem. Res.* 5 1–6. 10.15373/2249555x/august2014/189

[B143] Siloto R. M. Weselake R. J. (2012). Site saturation mutagenesis: Methods and applications in protein engineering. *Biocatal. Agric. Biotechnol.* 1 181–189. 10.1016/j.bcab.2012.03.010

[B144] Sousa M. Souza O. Maciel M. Cruz R. Rêgo M. G. Magalhães O. (2015). Keratinolytic potential of fungi isolated from soil preserved at the Micoteca URM. *Eur. J. Biotechnol. Biosci.* 3 10–15.

[B145] Srivastava B. Khatri M. Singh G. Arya S. K. (2020). Microbial keratinases: An overview of biochemical characterization and its eco-friendly approach for industrial applications. *J. Clean. Prod.* 252:119847 10.1016/j.jclepro.2019.119847

[B146] Stiborova H. Branska B. Vesela T. Lovecka P. Stranska M. Hajslova J. (2016). Transformation of raw feather waste into digestible peptides and amino acids. *J. Chem. Technol. Biotechnol.* 91 1629–1637. 10.1002/jctb.4912

[B147] Su C. Gong J. S. Zhang R. X. Tao L. Y. Dou W. F. Zhang D. D. (2017). A novel alkaline surfactant-stable keratinase with superior feather-degrading potential based on library screening strategy. *Int. J. Biol. Macromol.* 95 404–411. 10.1016/j.ijbiomac.2016.11.045 27864058

[B148] Suntornsuk W. Tongjun J. Onnim P. Oyama H. Ratanakanokchai K. Kusamran T. (2005). Purification and characterisation of keratinase from a thermotolerant feather-degrading bacterium. *World J. Microbiol. Biotechnol.* 21:1111 10.1007/s11274-005-0078-x

[B149] Takagi H. Morinaga Y. Ikemura H. Inouye M. (1988). Mutant subtilisin E with enhanced protease activity obtained by site-directed mutagenesis. *J. Biol. Chem.* 263 19592–19596. 3143728

[B150] Takenaka S. Miyatake A. Tanaka K. Kuntiya A. Techapun C. Leksawasdi N. (2015). Characterisation of the native form and the carboxy-terminally truncated halotolerant form of α-amylases from *Bacillus subtilis* strain FP-133. *J. Basic Microbiol.* 55 780–789. 10.1002/jobm.201400813 25689045

[B151] Tamreihao K. Mukherjee S. Khunjamayum R. Devi L. J. Asem R. S. Ningthoujam D. S. (2019). Feather degradation by keratinolytic bacteria and biofertilizing potential for sustainable agricultural production. *J. Basic Microbiol.* 59 4–13. 10.1002/jobm.201800434 30353928

[B152] Tatineni R. Doddapaneni K. K. Potumarthi R. C. Vellanki R. N. Kandathil M. T. Kolli N. (2008). Purification and characterisation of an alkaline keratinase from *Streptomyces* sp. *Bioresour. Technol.* 99 1596–1602. 10.1016/j.biortech.2007.04.019 17804219

[B153] Tesfaye T. Sithole B. Ramjugernath D. Chunilall V. (2017). Valorisation of chicken feathers: Characterisation of physical properties and morphological structure. *J. Clean Prod.* 149 349–365. 10.1016/j.jclepro.2017.02.112 28687152

[B154] Thankaswamy S. R. Sundaramoorthy S. Palanivel S. Ramudu K. N. (2018). Improved microbial degradation of animal hair waste from leather industry using *Brevibacterium luteolum* (MTCC 5982). *J. Clean. Prod.* 189 701–708. 10.1016/j.jclepro.2018.04.095

[B155] Thys R. C. S. Brandelli A. (2006). Purification and properties of a keratinolytic metalloprotease from *Microbacterium* sp. *J. Appl. Microbiol.* 101 1259–1268. 10.1111/j.1365-2672.2006.03050.x 17105556

[B156] Tian J. Xu Z. Long X. Tian Y. Shi B. (2019). High-expression keratinase by *Bacillus subtilis* SCK6 for enzymatic dehairing of goatskins. *Int. J. Biol. Macromol.* 135 119–126. 10.1016/j.ijbiomac.2019.05.131 31125653

[B157] Tiwary E. Gupta R. (2010). Medium optimisation for a novel 58 kDa dimeric keratinase from *Bacillus licheniformis* ER-15: biochemical characterisation and application in feather degradation and dehairing of hides. *Bioresour. Technol.* 101 6103–6110. 10.1016/j.biortech.2010.02.090 20347294

[B158] Tiwary E. Gupta R. (2012). Rapid conversion of chicken feather to feather meal using dimeric keratinase from *Bacillus licheniformis* ER-15. *J. Bioprocess. Biotech.* 2 1–5.

[B159] Toivola D. M. Boor P. Alam C. Strnad P. (2015). Keratins in health and disease. *Curr. Opin. Cell Biol.* 32 73–81. 10.1016/j.ceb.2014.12.008 25599598

[B160] Tsiroulnikov K. Rezai H. Bonch-Osmolovskaya E. Nedkov P. Gousterova A. Cueff V. (2004). Hydrolysis of the amyloid prion protein and nonpathogenic meat and bone meal by anaerobic thermophilic prokaryotes and *Streptomyces* subspecies. *J. Agric. Food Chem.* 52 6353–6360. 10.1021/jf0493324 15453713

[B161] ul Haq I. Akram F. (2018). Striking applications of keratinase enzyme isolated from various natural sources: a review. *Proc. Pakistan Acad. Sci B Life Environ. Sci.* 55 1–17.

[B162] Vasileva-Tonkova E. Gousterova A. Neshev G. (2009). Ecologically safe method for improved feather wastes biodegradation. *Int. Biodeterior. Biodegradation* 63 1008–1012. 10.1016/j.ibiod.2009.07.003

[B163] Verma A. Singh H. Anwar M. S. Kumar S. Ansari M. W. Agrawal S. (2016). Production of thermostable organic solvent tolerant keratinolytic protease from *Thermoactinomyces* sp. RM4: IAA production and plant growth promotion. *Front. Microbiol.* 7:1189. 10.3389/fmicb.2016.01189 27555836 PMC4974946

[B164] Verma A. Singh H. Anwar S. Chattopadhyay A. Tiwari K. K. Kaur S. (2017). Microbial keratinases: industrial enzymes with waste management potential. *Crit. Rev. Biotechnol.* 37 476–491. 10.1080/07388551.2016.1185388 27291252

[B165] Vidmar B. Vodovnik M. (2018). Microbial keratinases: enzymes with promising biotechnological applications. *Food Technol. Biotechnol.* 56 312–328. 30510475 10.17113/ftb.56.03.18.5658 PMC6233012

[B166] Villa A. L. V. Aragão M. R. S. dos Santos E. P. Mazotto A. M. Zingali R. B. de Souza E. P. (2013). Feather keratin hydrolysates obtained from microbial keratinases: effect on hair fiber. *BMC Biotechnol.* 13:15. 10.1186/1472-6750-13-15 23414102 PMC3621039

[B167] Vojcic L. Pitzler C. Koerfer G. Jakob F. Martinez R. Maurer K. H. (2015). Advances in protease engineering for laundry detergents. *New Biotechnol.* 32 629–634. 10.1016/j.nbt.2014.12.010 25579194

[B168] Wang B. Yang W. McKittrick J. Meyers M. A. (2016). Keratin: Structure, mechanical properties, occurrence in biological organisms, and efforts at bioinspiration. *Prog. Mater. Sci.* 76 229–318. 10.1016/j.pmatsci.2015.06.001

[B169] Wang F. Zieman A. Coulombe P. A. (2016). Skin keratins. *Methods Enzymol.* 568 303–350. 26795476 10.1016/bs.mie.2015.09.032 PMC4902878

[B170] Wang J. J. Garlich J. D. Shih J. C. H. (2006). Beneficial effects of versazyme, a keratinase feed additive, on body weight, feed conversion, and breast yield of broiler chickens. *J. Appl. Poult. Res.* 15 544–550. 10.1093/japr/15.4.544

[B171] Wells J. A. Cunningham B. C. Graycar T. P. Estell D. A. (1987). Recruitment of substrate-specificity properties from one enzyme into a related one by protein engineering. *Proc. Natl. Acad. Sci. U.S.A.* 84 5167–5171. 10.1073/pnas.84.15.5167 3299378 PMC298815

[B172] Williams C. M. Lee C. G. Garlich J. D. Shih J. C. (1991). Evaluation of a bacterial feather fermentation product, feather-lysate, as a feed protein. *Poult. Sci.* 70 85–94. 10.3382/ps.0700085

[B173] Williams C. M. Richter C. S. Mackenzie J. M. Shih J. C. (1990). Isolation, identification, and characterisation of a feather-degrading bacterium. *Appl. Environ. Microbiol.* 56 1509–1515. 10.1128/aem.56.6.1509-1515.1990 16348199 PMC184462

[B174] Wu W. L. Chen M. Y. Tu I. F. Lin Y. C. EswarKumar N. Chen M. Y. (2017). The discovery of novel heat-stable keratinases from *Meiothermus taiwanensis* WR-220 and other extremophiles. *Sci. Rep.* 7 1–12. 10.1007/s00792-009-0281-z 28680127 PMC5498600

[B175] Xia Y. Massé D. I. McAllister T. A. Beaulieu C. Ungerfeld E. (2012). Anaerobic digestion of chicken feather with swine manure or slaughterhouse sludge for biogas production. *Waste Manage.* 32 404–409. 10.1016/j.wasman.2011.10.024 22088961

[B176] Yamamura S. Morita Y. Hasan Q. Yokoyama K. Tamiya E. (2002). Keratin degradation: a cooperative action of two enzymes from *Stenotrophomonas* sp. *Biochem. Biophys. Res. Commun.* 294 1138–1143. 10.1016/s0006-291x(02)00580-6 12074595

[B177] Yan B. Q. Chen X. L. Hou X. Y. He H. Zhou B. C. Zhang Y. Z. (2009). Molecular analysis of the gene encoding a cold-adapted halophilic subtilase from deep-sea psychrotolerant bacterium *Pseudoalteromonas* sp. SM9913: cloning, expression, characterisation and function analysis of the C-terminal PPC domains. *Extremophiles* 13 725–733. 10.1007/s00792-009-0263-1 19544039

[B178] Yang H. Li J. Du G. Liu L. (2017). “Microbial production and molecular engineering of industrial enzymes: challenges and strategies,” in *Biotechnology of Microbial Enzymes*, ed. Brahmachari G. (Cambridge, MA: Academic Press), 151–165. 10.1016/b978-0-12-803725-6.00006-6

[B179] Zhang R. X. Gong J. S. Su C. Zhang D. D. Tian H. Dou W. F. (2016). Biochemical characterisation of a novel surfactant-stable serine keratinase with no collagenase activity from *Brevibacillus parabrevis* CGMCC 10798. *Int. J. Biol. Macromol.* 93 843–851. 10.1016/j.ijbiomac.2016.09.063 27651275

[B180] Zhang X. (2012). Applying the mutation of *Bacillus subtilis* and the optimisation of feather fermentation medium to improve Keratinase activity. *Adv. Biol. Chem.* 2:6 10.4236/abc.2012.21008

[B181] Zhang Z. Li D. Zhang X. (2019). Enzymatic decolorization of melanoidins from molasses wastewater by immobilized keratinase. *Bioresour. Technol.* 280 165–172. 10.1016/j.biortech.2019.02.049 30771571

[B182] Zhao H. Y. Feng H. (2018). Engineering *Bacillus pumilus* alkaline serine protease to increase its low-temperature proteolytic activity by directed evolution. *BMC Biotechnol.* 18:34. 10.1186/s12896-018-0451-0 29859069 PMC5984802

